# Genetic analysis of isoform usage in the human anti-viral response reveals influenza-specific regulation of *ERAP2* transcripts under balancing selection

**DOI:** 10.1101/gr.240390.118

**Published:** 2018-12

**Authors:** Chun Jimmie Ye, Jenny Chen, Alexandra-Chloé Villani, Rachel E. Gate, Meena Subramaniam, Tushar Bhangale, Mark N. Lee, Towfique Raj, Raktima Raychowdhury, Weibo Li, Noga Rogel, Sean Simmons, Selina H. Imboywa, Portia I. Chipendo, Cristin McCabe, Michelle H. Lee, Irene Y. Frohlich, Barbara E. Stranger, Philip L. De Jager, Aviv Regev, Tim Behrens, Nir Hacohen

**Affiliations:** 1 Institute for Human Genetics, Institute for Health and Computational Sciences, Department of Biostatistics and Epidemiology, Department of Bioengineering and Therapeutic Sciences, University of California, San Francisco, California 94143, USA;; 2 Broad Institute of MIT and Harvard, Cambridge, Massachusetts 02142, USA;; 3 Division of Health Sciences and Technology, Massachusetts Institute of Technology, Cambridge, Massachusetts 02139, USA;; 4 Department of Medicine, Massachusetts General Hospital Cancer Center, Boston, Massachusetts 02114, USA;; 5 Biomedical Informatics Program, University of California, San Francisco, California 94143, USA;; 6 Genentech Incorporated, South San Francisco, California 94080, USA;; 7 Harvard Medical School, Boston, Massachusetts 02116, USA;; 8 Departments of Neurology and Psychiatry, Brigham and Women's Hospital, Boston, Massachusetts 02115, USA;; 9 Section of Genetic Medicine, Department of Medicine, Institute for Genomics and Systems Biology, Center for Data Intensive Science, The University of Chicago, Chicago, Illinois 60637, USA;; 10 Department of Biology, Massachusetts Institute of Technology, Cambridge, Massachusetts 02139, USA;; 11 Howard Hughes Medical Institute, Chevy Chase, Maryland 20815, USA

## Abstract

While genetic variants are known to be associated with overall gene abundance in stimulated immune cells, less is known about their effects on alternative isoform usage. By analyzing RNA-seq profiles of monocyte-derived dendritic cells from 243 individuals, we uncovered thousands of unannotated isoforms synthesized in response to influenza infection and type 1 interferon stimulation. We identified more than a thousand quantitative trait loci (QTLs) associated with alternate isoform usage (isoQTLs), many of which are independent of expression QTLs (eQTLs) for the same gene. Compared with eQTLs, isoQTLs are enriched for splice sites and untranslated regions, but depleted of sequences upstream of annotated transcription start sites. Both eQTLs and isoQTLs explain a significant proportion of the disease heritability attributed to common genetic variants. At the *ERAP2* locus, we shed light on the function of the gene and how two frequent, highly differentiated haplotypes with intermediate frequencies could be maintained by balancing selection. At baseline and following type 1 interferon stimulation, the major haplotype is associated with low *ERAP2* expression caused by nonsense-mediated decay, while the minor haplotype, known to increase Crohn's disease risk, is associated with high *ERAP2* expression. In response to influenza infection, we found two uncharacterized isoforms expressed from the major haplotype, likely the result of multiple perfectly linked variants affecting the transcription and splicing at the locus. Thus, genetic variants at a single locus could modulate independent gene regulatory processes in innate immune responses and, in the case of *ERAP2*, may confer a historical fitness advantage in response to virus.

An important aspect of eukaryotic gene regulation is the usage of alternative gene isoforms. This is achieved through several mechanisms at the transcript level, including alternative promoters for transcription initiation, alternative splicing of pre-messenger RNA, alternative polyadenylation, and selective degradation of isoforms. These processes regulate the relative abundances of multiple coding and noncoding RNAs from the same underlying DNA sequence, often resulting in altered function of the gene products in response to developmental or environmental changes (Black 2003; Maquat 2004; Matlin et al. 2005; Wang et al. 2008).

A case in point is the critical role of alternative isoform usage across many immune processes, such as the balance between IgM and IgD immunoglobulin isoforms in B cells (Enders et al. 2014), naïve and memory T-cell proportions controlled by *PTPRC* isoforms (Berard and Tough 2002), and innate immune responses to pathogens regulated by different isoforms of *MYD88* (Martinez and Lynch 2013). Genetic variants that affect isoform usage have been associated with immune disorders (Xiong et al. 2015), including single-nucleotide polymorphisms (SNPs) that alter the relative splicing of two *IRF5* isoforms (Graham et al. 2007) associated with systemic lupus erythematosus (SLE).

Previous studies have identified shared and divergent transcriptional programs in the antibacterial and antiviral responses of innate immune cells (Amit et al. 2009; Lee et al. 2014), with genetic variants imparting stimulation-specific effects on the total transcript abundances of thousands of genes (Barreiro et al. 2012; Fairfax and Knight 2014; Lee et al. 2014; Quach et al. 2016). While maps of genetic determinants of alternative isoform usage are beginning to emerge, most notably in lymphoblastoid cell lines (Lappalainen et al. 2013; Li et al. 2016), across post-mortem human tissues (The GTEx Consortium 2015; Rivas et al. 2015), and in macrophages stimulated with bacteria (Nedelec et al. 2016), differential isoform usage in the human antiviral response, its natural variability, and its genetic basis have not been studied.

Here, we integrate bulk RNA-sequencing with dense genotyping to systematically investigate the genetic control of isoform usage in monocyte-derived dendritic cells (MoDCs) at rest, and in response to influenza infection or type 1 interferon stimulation. Because the type 1 interferon pathway is known to be engaged by a broad array of microbial products, our study design is unique in allowing the separation of the universal and influenza-specific interferon-induced responses. Since the human transcriptome has never been annotated under these conditions, we first used de novo assembly to catalog and expectation maximization to quantify all synthesized isoforms in resting and stimulated MoDCs. Then, by harnessing the natural transcriptomic and genetic variation in the ImmVar cohort (Lee et al. 2014; Raj et al. 2014a; Ye et al. 2014), we mapped quantitative trait loci (QTLs) associated with alternate isoform usage (isoQTLs). Systematic characterization of isoQTLs, especially in comparison to expression quantitative trait loci (eQTLs), provides mechanistic insights into the genetic control of different aspects of gene regulation and enables the functional interpretation of loci associated with immune-related diseases and under natural selection.

## Results

### Influenza infection and type I interferon stimulation induce widespread alternate isoform usage

We used paired-end RNA-seq to profile the transcriptomes of primary MoDCs from healthy donors at rest (*N* = 99), and following stimulation with either recombinant interferon beta (IFNB1), a type 1 interferon that stimulates anti-viral effectors (*N* = 227), or influenza ΔNS1 (a strain engineered to maximize the type 1 interferon-induced response to infection by the deletion of a key virulence factor; *N* = 250) (Shapira et al. 2009). A total of 552 pass-filter samples (out of 576), 84 from all three conditions, 127 from both stimulation conditions, and 46 from only one condition, were analyzed (Supplemental Table S1). To define the corpus of transcribed isoforms in human MoDCs at rest and in response to stimulation, we assembled the transcriptome de novo in each sample (individual-condition pair) from RNA-seq alignments, retained only expressed isoforms (more than five transcripts per million [TPM] in any sample), and then combined isoforms across all samples to enable direct comparisons between conditions. We noted that unannotated isoforms that did not match current GENCODE, UCSC, or RefSeq annotations (Harrow et al. 2006; O'Leary et al. 2016; Casper et al. 2018) were enriched in genes expressed at less than 25 TPMs across all three conditions (Supplemental Fig. S1), and thus removed these genes and their corresponding transcripts from downstream analyses.

Our final assembled transcriptome contained 15,754 transcripts corresponding to 8194 genes (Supplemental Table S2), 64.5% of which had transcriptional structures that exactly matched an annotated transcript; 5204 transcripts (33.0%) contained a novel splice site, and 389 transcripts (2.5%) did not harbor novel splice sites but contained novel splice junctions. These novel splice events were well supported by the presence of spliced reads (more than 10 mapped reads) spanning exon junctions of our assembled transcriptome. Of the 25,099 novel splice events, 11,093 (44%) were within 5′ UTRs and 7251 (28.9%) were within 3′ UTRs, echoing a previous deep sequencing analysis that reported that the majority of novel isoforms are due to alternative splicing of UTRs (Deveson et al. 2018). An additional 2663 transcripts (16.9%) were annotated with a novel transcription start site (TSS). To assess the accuracy of these new TSSs, we aligned CAGE sequencing reads from resting MoDCs (Noguchi et al. 2017) to the set of unique TSSs (TSS + 500 bp downstream) from assembled transcripts in the baseline condition. Compared with all TSSs, 69% (vs. 84%) of the new TSSs were supported by the presence of at least one mapped CAGE read, and 40% (vs. 61%) were supported by more than five mapped reads. By visual inspection, we found the most common misannotations to be isoform reconstructions that began within the gene body, downstream from the annotated TSS. While some of these fragments may truly exist, we were unable to verify them with short read sequencing and thus conservatively estimated the false annotation rate to be ∼20% of new TSSs (difference between new and all TSSs) or 3.3% of our entire transcriptome.

**Figure 1.**
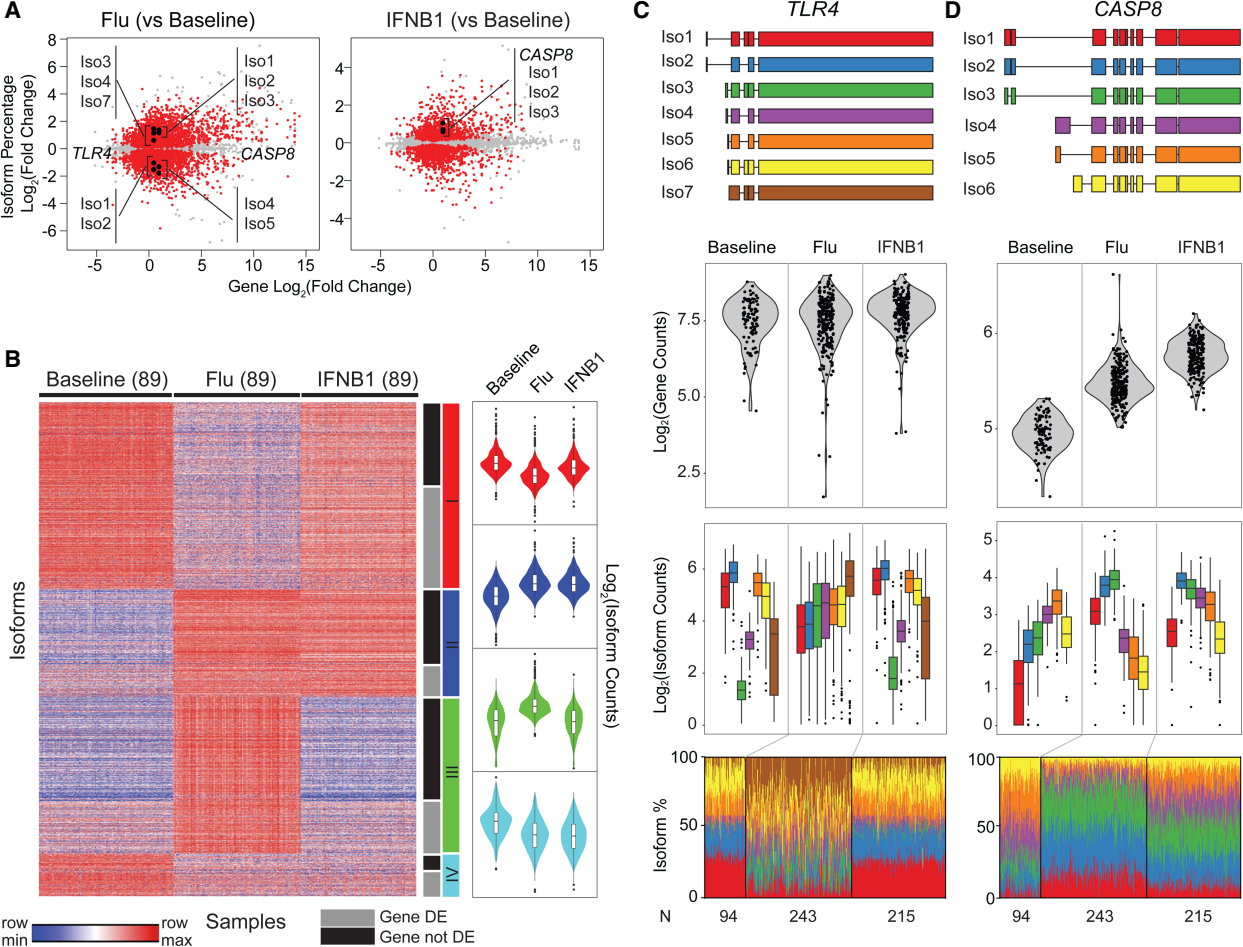
Transcriptome changes in response to stimulation. (*A*) Scatter plot of log_2_ fold change of gene abundance (*x*-axis) versus log_2_ fold change of isoform usage percentage (*y*-axis) in flu-infected (*left*) and IFNB1-simulated (*right*) cells compared with baseline. Each dot represents one isoform. Isoforms that significantly differed in their usage (beta regression, FDR < 0.05) are highlighted in red. (*B*) Clustering of isoform usage ratios in baseline, flu-infected, and IFNB1-stimulated cells. Heatmap colors are row scaled (red indicates row maximum, blue indicates row minimum; *left*). Violin plots (*right*) summarize the usages of all isoforms within a cluster separated by condition. Only isoforms (one per gene) that most significantly changed (beta regression, FDR < 0.05) in usage are shown. (*C*,*D*) De novo constructed isoforms (*top*), gene abundance (*second* row), and isoform abundance (*third* row) and usage percentage (*bottom*) for *TLR4* (*C*) and *CASP8* (*D*).

We next compared changes in isoform usage—estimated as the ratio of isoform abundance over the total gene abundance—in response to each stimulus. Relative to baseline, the usages of approximately twice as many isoforms (5326 vs. 2509) were altered in flu-infected compared with IFNB1-stimulated cells (beta regression, FDR < 0.05) (Supplemental Table S3; Supplemental Fig. S1A). This was corroborated by directly comparing flu-infected and IFNB1-stimulated cells where the usages of 5072 isoforms were altered (beta regression, FDR < 0.05) (Supplemental Table S3; Supplemental Fig. S2). In response to either stimulus, more than a third of the isoforms with differential usage were previously unannotated, highlighting the inadequacy of current transcriptome annotations in describing the full repertoire of gene isoforms in the human antiviral response. Of the differentially expressed genes (DESeq; FDR < 0.05, gene abs(log_2_[fold change]) > 1) (Supplemental Table S4) with more than one isoform, 54% (2233/4120) in flu-infected cells and 29% (1122/3898) in IFNB1-stimulated cells had at least one isoform that differed in usage (Fig. 1A).

Isoforms that differed in usage partitioned into four prominent clusters (Fig. 1B, *k*-means clustering of the most significant isoforms, one per gene; Supplemental Table S5). Isoforms with increased usage in response to both stimuli (Cluster II) were highly enriched for innate system processes (GO:0002376, *q* < 6.41 × 10^−6^), defense response to virus (GO:0051607, *q* < 9.56 × 10^−6^), and type 1 interferon signaling pathway (GO:0060337, *q* < 1.25 × 10^−3^). Isoforms with increased usage in response to flu but not IFNB1 (Cluster III) were enriched for regulators of gene expression (GO:0010468, *q* < 6.23 × 10^−3^), including genes involved in the regulation of MAP kinase cascade (GO:0043408, *q* < 1.13 × 10^−2^) and inflammatory response (GO:0006954, *q* < 1.88 × 10^−2^). Isoforms with decreased usage in response to flu (Cluster I) were enriched for oxidoreductase activity (GO:0016616, *q* < 2.47 × 10^−2^); isoforms with decreased usage in response to both conditions (Cluster IV) were not significantly enriched for known Gene Ontology entries. *TLR4*, the toll-like receptor classically associated with sensing bacterial ligands but also shown to sense viral products (Doyle et al. 2002), was among the genes that had flu-specific isoform usage despite little change in total gene abundance (Fig. 1C; Supplemental Fig. S3). In flu-infected cells, the usages of longer isoforms with an upstream alternative start site (*TLR4*/Iso1 and *TLR4*/Iso2) were decreased, while the usages of *TLR4*/Iso3, *TLR4*/Iso4, and *TLR4*/Iso7 were increased. *TLR4*/Iso4 encodes the annotated 839-amino-acid product, while isoforms *TLR4*/Iso3 and *TLR4*/Iso7 encode shorter, 799-amino-acid products each with a truncated extracellular domain missing a predicted signal peptide. We also found decreased usage of short *CASP8* isoforms (*CASP8*/Iso4, *CASP8*/Iso5, *CASP8*/Iso6) (Fig. 1D; Supplemental Fig. S3) only in flu-infected cells. *CASP8* is best known to induce apoptosis via the Fas-associated protein with death domain (FADD) in response to extrinsic cytokine signals. *CASP8*/Iso4 has a unique N-terminal extension of 59 amino acids, which has been reported to allow for selective recruitment to the endoplasmic reticulum (Breckenridge et al. 2002). These results demonstrate that changes in isoform usage independent of overall gene abundance are pervasive and affect prominent innate immune sensors and regulators in viral versus interferon response.

### Genetic variants associated with isoform usage are enriched for distinct gene regulatory elements

While it is known that common genetic variants modulate gene expression in both resting and stimulated MoDCs (Lee et al. 2014), we assessed if they could also affect isoform usage under these conditions. We associated over 10 million imputed variants with two transcriptional traits, isoform usage ratio and log of total gene abundance, to identify isoQTLs and eQTLs, respectively. After adjusting for unwanted variation that likely tracked with technical and biological confounders (Supplemental Figs. S4, S5), we identified 2763 isoforms corresponding to 1425 genes (linear regression, permutation FDR < 0.05) (Supplemental Table S6) with local isoQTLs (±500 kb of TSS) and 6694 genes (linear regression, permutation FDR < 0.05) (Supplemental Table S7) with local eQTLs in at least one condition. A substantial proportion of leading isoQTL SNPs (63% baseline, 40% flu, 41% IFNB1) were not significant eQTLs, suggesting that the genetic control of isoform usage and overall gene abundance are largely independent (Methods; Supplemental Figs. S6, S7).

**Figure 2.**
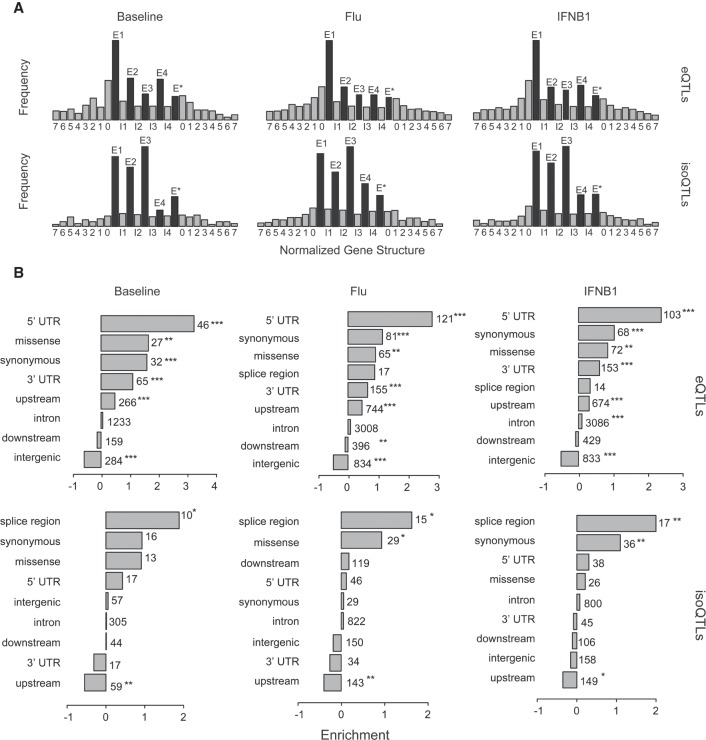
Properties of local eQTLs and isoQTLs. (*A*) Frequency (*y*-axis) of the location of leading SNPs for local eQTLs and isoQTLs (permutation FDR < 0.05) with respect to meta gene structure (*x*-axis). Genes are normalized to five exonic regions (E1–E4 indicate exons 1 through 4; E* indicates exon 5 to the last exon) and four intronic regions (introns 1, 2, 3, and from intron 4 to the last intron). Upstream and downstream sequences are divided into 100-kb windows. (*B*) Log_2_ fold enrichment (*x*-axis) of leading SNPs for local eQTLs and isoQTLs (permutation FDR < 0.05) for genomic annotations. eQTL enrichments are calculated using a background set of SNPs matched for distance to TSS and allele frequency. IsoQTL enrichments are calculated with respect to a background set of eQTLs matched for distance to TSS and allele frequency. Multiple testing significance is indicated. (*) *P* < 0.05; (**) *P* < 0.01; (***) *P* < 0.001.

Genetic variants could modulate isoform usage through several mechanisms, including perturbing the usage of alternate promoters, splice sites, or regulatory elements in the UTRs. We compared the genomic properties of isoQTLs and eQTLs to identify the mechanisms by which each class of variants acts. When normalized by exon and intron lengths, leading SNPs for local isoQTLs (one per isoform) were enriched across the entire gene body (Fig. 2A), in distinct contrast to leading SNPs for local eQTLs (one per gene), which were enriched near TSSs and transcription end sites (TESs). Further, compared with a set of eQTLs matched for allele frequency and distance to TSS, leading SNPs for local isoQTLs were most enriched for splice sites (baseline: 3.7×, flu: 3.0×, IFNB1: 4.0×), synonymous (baseline: 1.9×, IFNB1: 2.1×) and missense variants (baseline: 1.9×, flu: 1.8×), and 5′ UTRs (baseline: 1.3×, IFNB1: 1.2×) (Fig. 2B). Compared with eQTLs, isoQTLs were not enriched for binding sites of key transcription factors involved in myeloid cell response (Supplemental Fig. S8). These results suggest that genetic variants associated with isoform usage likely do so via *cis* regulatory sequences that modulate alternative splicing and transcript stability.

### Genetic control of alternative isoform usage in responses to virus infection and IFNB1 stimulation

**Figure 3.**
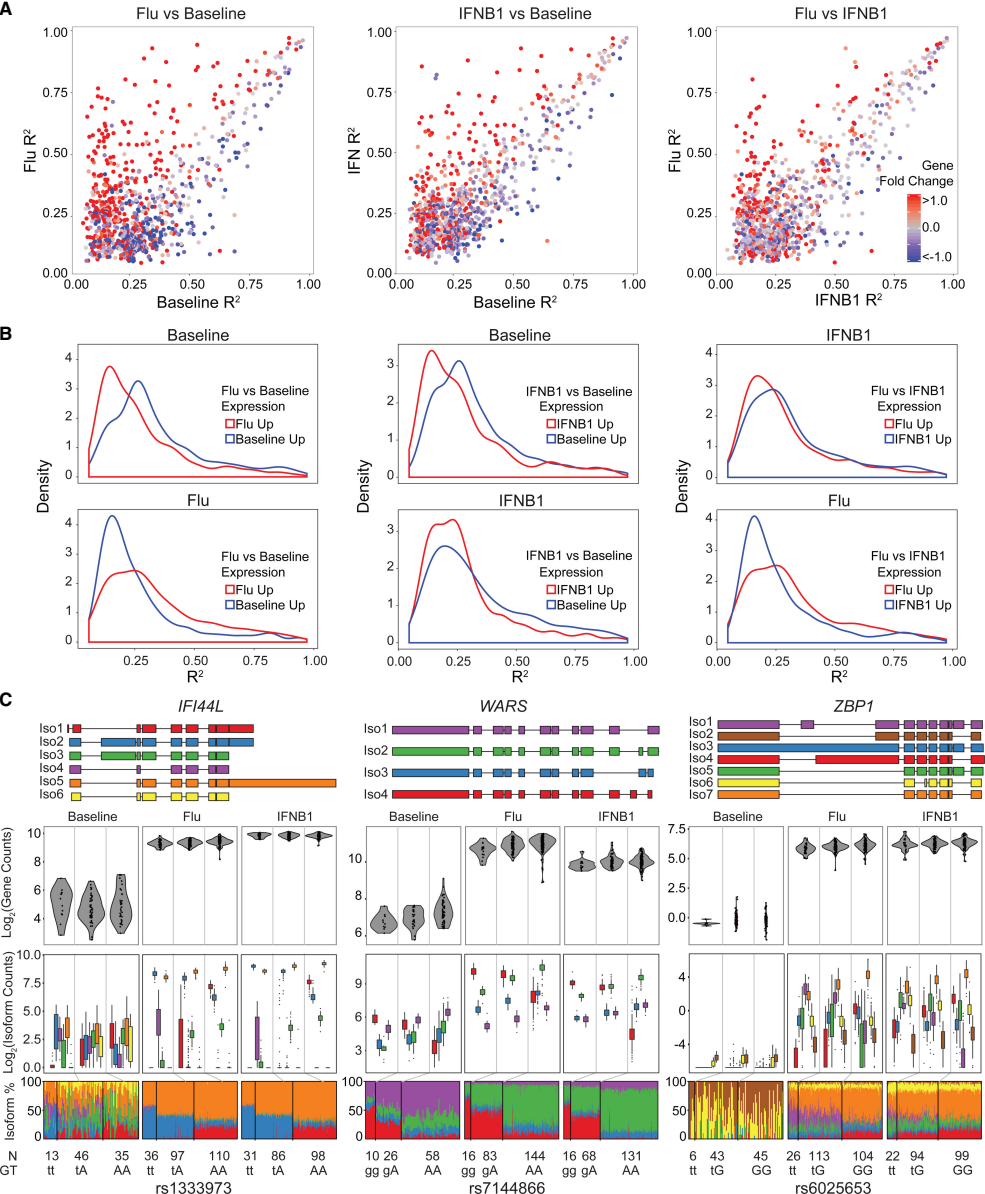
Comparison of local isoQTLs between conditions. (*A*) Correlation of effect sizes (*R*^2^) for significant local isoQTLs (permutation FDR < 0.05) between pairs of conditions. Transcripts are colored by differential expression (red indicates up-regulated in condition 2, *y*-axis; blue indicates up-regulated in condition 1, *x*-axis) for each pair of conditions. (*B*) Distributions of effect sizes (*R*^2^) for significant local isoQTLs (permutation FDR < 0.05) for each pair of conditions segregating genes based on expression in each condition. (*C*) De novo constructed transcript structure (*top*) and box-whisker plots (*bottom* three panels) between transcript quantitative traits (*y*-axis: log_2_(normalized gene abundance), log_2_(normalized isoform abundance), or isoform usage percentage) and genotype (*x*-axis) for three genes (*IFI44L*, *WARS*, and *ZBP1*) with risoQTLs.

To assess how the genetic control of isoform usage differs in response to stimuli, we analyzed 84 donors whose cells were assayed in all three conditions to enable equally powered comparisons. At these sample sizes, we detected more eQTLs in cells stimulated with IFNB1 than in cells at rest or infected with flu (baseline: 1715, flu: 1755, IFNB1: 2108; permutation FDR < 0.05) (Supplemental Table S6) but similar numbers of isoQTLs across conditions (baseline: 717, flu: 644, IFNB1: 692; permutation FDR < 0.05) (Supplemental Table S7). For the 1164 isoforms with isoQTLs in at least one condition, we compared the effect sizes (R_iso_^2^) of associations across conditions (Fig. 3A). The correlation of R_iso_^2^s was lowest between flu-infected and resting (baseline) cells (Pearson's *r*_flu.baseline_ = 0.61 compared with *r*_IFNB1.baseline_ = 0.76 and *r*_IFNB1.flu_ = 0.74). The corresponding genes of isoforms with higher R_iso_^2^ in stimulated cells were up-regulated in response to stimuli, suggesting that the genetic control of isoform usage is sensitive to activation of gene regulatory programs that control overall gene abundance (Fig. 3A,B).

To directly assess how stimulation modifies the effects of genetic variants on isoform usage, we mapped SNPs associated with the difference in isoform usage between conditions, herein referred to as local response-isoQTLs (risoQTLs). We identified, compared to resting cells, 53 (flu) and 30 (IFNB1) isoforms, corresponding to 31 and 14 genes, with at least one local risoQTLs (permutation FDR < 0.05) (Supplemental Table S8). Among the seven genes that shared local risoQTLs in both stimulated conditions were *IFI44L* and *WARS* (Fig. 3C). *IFI44L* is a type 1 interferon-stimulated gene known to moderately inhibit human hepatitis virus replication in vitro (Schoggins et al. 2011) and whose splicing has been shown to be influenced by rs1333973 (Lalonde et al. 2011). Rs1333973 is the most significant risoQTL associated with the usages of two isoforms differentiated by exon 2 in flu-infected and IFNB1-stimulated cells (flu vs. baseline: *IFI44L*/Iso1: +8.9%, *P* < 1.37 × 10^−9^; *IFI44L*/Iso2: −13.9%, *P* < 1.53 × 10^−9^; IFNB1 vs. baseline: *IFI44L*/Iso1: +9.6%, *P* < 1.21 × 10^−10^; *IFI44L*/Iso2: −15.7%, *P* < 2.14 × 10^−10^) (Supplemental Fig. S9). *WARS* is a tryptophanyl-tRNA synthetase primarily involved in protein synthesis. While *WARS* isoforms are known to encode for catalytic null enzymes (Lo et al. 2014) and have anti-angiogenic activity inducible by interferon gamma (Wakasugi et al. 2002), there have been no previous reports of the genetic control of these isoforms. The most significant risoQTL rs7144866 was associated with the usages of two isoforms differentiated by exon 2: While rs7144866^A^ increases the usage of *WARS*/Iso1 in cells at rest, it increases the usage of *WARS*/Iso2 in flu-infected and IFNB1-stimulated cells resulting in the risoQTL (flu vs. baseline: −19.1%, 2.50 × 10^−29^; IFNB1 vs. baseline: −17.7%, 7.03 × 10^−26^) (Supplemental Fig. S10). There were 40 isoforms, corresponding to 30 genes, that have at least one risoQTL in flu-infected cells compared with interferon-stimulated cells (permutation FDR < 0.05) (Supplemental Table S8). Among these was *ZBP1*, a sensor of influenza infection that triggers cell death and inflammation and contributes to virus-induced lethality (Kuriakose et al. 2016). We found rs6025653^t^ increases the usage of *ZBP1*/Iso1 by 9.67% (*P* < 4.16 × 10^−16^) (Fig. 3C; Supplemental Fig. S11) in flu-infected compared with interferon-stimulated cells. *ZBP1*/Iso1 is differentiated from all other *ZBP1* isoforms by the retention of exon 9. These results suggest that while influenza-infected and interferon-stimulated cells expectedly share some genetic control of isoform usage (as interferons are induced by viral infection), influenza infection also confers specific genetic control of isoform usage of previously unknown genes, likely reflecting antiviral mechanisms independent of downstream effector (type 1 interferon) signaling.

### Association of eQTLs and isoQTLs with immune-related diseases

**Figure 4.**
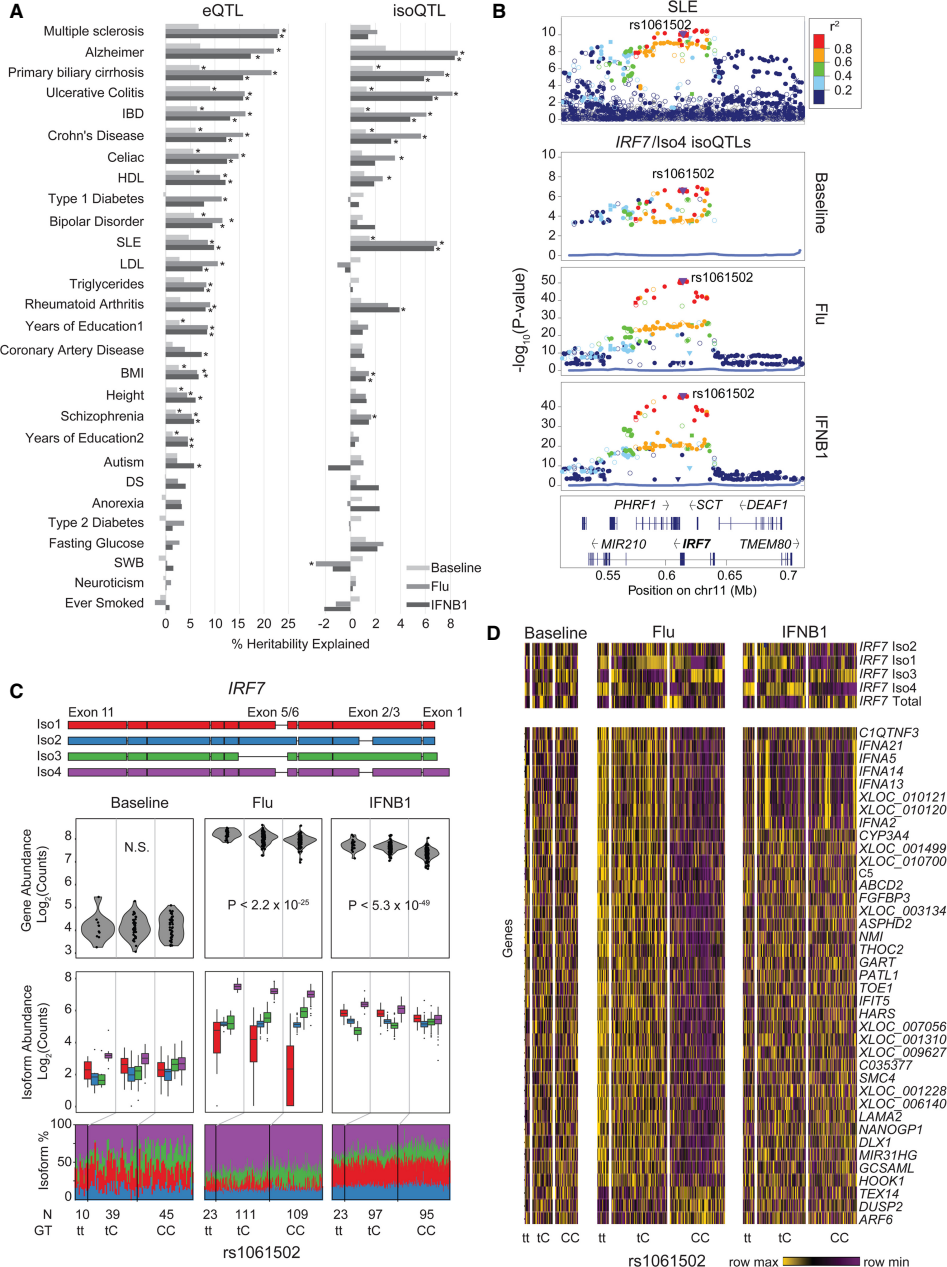
GWAS enrichment of local eQTLs and isoQTLs. (*A*) Partitioned heritability analysis: proportion of SNP heritability explained (*x*-axis) for 28 traits (*y*-axis) by eQTLs (*left*) and isoQTLs (*right*). (DS) Depressive symptoms; (SWB) subject well-being; (IBD) inflammatory bowel disease; (SLE) systemic lupus erythematosus; (BMI) body mass index; (HDL) high density lipoprotein; (LDL) low density lipoprotein. (*B*) LocusZoom plots of the *IRF7* region for SLE GWAS associations (*top*) and *IRF7*/Iso4 isoQTLs for baseline, flu-infected, or IFNB1-stimulated cells (*bottom* three panels). (*y*-axis) −log_10_(*P*-value) of association; (*x*-axis) genomic location. Points are colored based on LD to rs1061502. (*C*) Transcript structure (*top*) and box-whisker plots (*bottom* three panels) between *IRF7* transcript quantitative traits (*y*-axis: log_2_(normalized gene abundance), log_2_(normalized isoform abundance), or isoform usage percentage) and rs1061502 genotype (*x*-axis). (*D*) Heatmap of genes distally associated (permutation FDR < 0.05) with risoQTL rs1061502. Heatmap colors are row-scaled TPM values (yellow indicates row maximum; purple indicates row minimum).

Previous analyses of the overlap between expression QTLs and genome-wide association studies (GWAS) have aided the localization and functional interpretation of causal variants at GWAS loci. Because disease-causing variants that affect isoform usage could have more profound effects on gene regulation by altering protein structure, we jointly analyzed disease-associated variants and local isoQTLs in addition to local eQTLs using two approaches. First, compared with SNPs from the latest GWAS catalog (MacArthur et al. 2017), local eQTLs in stimulated cells were enriched in multiple diseases, including inflammatory bowel disease (flu: *P* < 5.46 × 10^−7^, IFNB1: *P* < 5.21 × 10^−5^), rheumatoid arthritis (flu: *P* < 2.49 × 10^−5^, IFNB1: *P* < 0.03), and Parkinson's disease (flu: *P* < 4.06 × 10^−7^, IFNB1: *P* < 7.44 × 10^−5^) (Supplemental Fig. S12), while local isoQTLs were enriched in late onset Alzheimer's disease (flu: *P* < 1.15 × 10^−6^, IFNB1: *P* < 1.75 × 10^−4^), vitiligo (flu: *P* < 4.94 × 10^−5^, IFNB1: *P* < 0.42), and SLE (flu: *P* < 0.1, IFNB1: *P* < 3.62 × 10^−3^) (Supplemental Fig. S12). Notably, the overlap of isoQTLs with Alzheimer's loci included genes with known variants that affect splicing: *CD33* (Hernandez-Caselles et al. 2006; Raj et al. 2014b) and *CD46* (Russell et al. 1992). Corroborating this, we performed partitioned heritability analysis using linkage disequilibrium (LD) score regression (Finucane et al. 2015). For 28 traits with available summary statistics, both local eQTLs and local isoQTLs explained a statistically significant percentage of the SNP heritability of autoimmune diseases (e.g., ulcerative colitis, SLE) and some neurodegenerative diseases (e.g., Alzheimer's) and did not explain much of the SNP heritability of diseases with no known relationship to the innate immune system (e.g., type 2 diabetes) (Fig. 4A; Supplemental Table S9). These results suggest a role for both variants that affects isoform usage and gene expression in mediating autoimmune and neurodegenerative disease risk.

The *IRF7* locus harbors an isoQTL within an extended haplotype previously known to be associated with SLE (rs58688157 lead SNP, *P* < 2.97 × 10^−11^) (Fig. 4B; Morris et al. 2016). A linked SNP (rs1061502; LD *R*^2^ = 0.93, *D*′ = 0.97 to rs58688157) was the most significant association to overall *IRF7* expression in IFNB1-stimulated (*P* < 2.87 × 10^−49^) and flu-infected cells (*P* < 2.21 × 10^−25^) (Fig. 4C; Supplemental Fig. S13). IsoQTL analysis further revealed that rs1061502^T^ also increased the usage of *IRF7*/Iso4 (flu: beta = 5.3%, *P* < 9.42 × 10^−52^; IFNB1: beta = 5.8%, *P* < 4.81 × 10^−46^) (Fig. 4C, panel 3, purple; Supplemental Fig. S14) while it decreased the usage of *IRF7*/Iso3 (flu: beta = −3.4%, 1.15 × 10^−15^; IFNB1: beta = −7.0%, 2.04 × 10^−29^) (Fig. 4C, panel 3, green; Supplemental Fig. S14). Further, although overall *IRF7* abundances were similar between the two stimulated conditions, *IRF7*/Iso4 (purple) was the dominant isoform in flu-infected cells but not in IFNB1-stimulated cells (10.7× fold, *P* < 10^−306^) (Fig. 4C, bottom panel; Supplemental Fig. S14). Rs1061502 was also a distal eQTL (permutation FDR < 0.2) for a cluster of genes, including *NMI*, *IFNA2*, *IFIT5*, and *C5*, only in flu-infected but not in IFNB1-stimulated cells (Fig. 4D). These results replicate and expand our previous findings that rs12805435 (LD *R*^2^ = 0.95, *D*′ = 0.98 to rs1061502) is associated in *cis* with *IRF7* expression in IFNB1-stimulated and flu-infected cells and in *trans* with a cluster of *IRF7-*regulated genes only in flu-infected cells (Lee et al. 2014). The flu-specific *trans* associations could be due to the additive effects of flu-specific induction of *IRF7*/Iso4 independent of IFNB1 signaling and induction of overall *IRF7* expression by rs1061502^T^. *IRF7*/Iso4 encodes a 516-amino-acid protein product and differs from other abundant isoforms in IFNB1-stimulated cells (*IRF7*/Iso1 and *IRF7*/Iso3) in the 5′ UTR and the coding sequence in the DNA-binding domain. Given the known link between type 1 interferons and SLE, our results suggest that SNPs affecting a specific *IRF7* isoform could impact viral responses and autoimmune inflammation through similar mechanisms.

### An *ERAP2* risoQTL controls differential transcript usage during influenza infection

**Figure 5.**
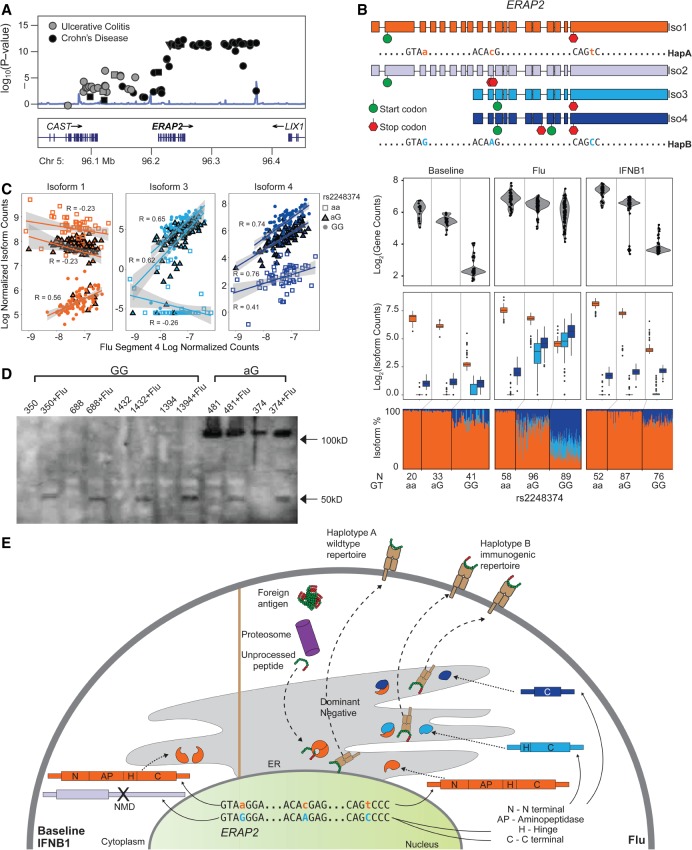
Genetics of *ERAP2* regulation. (*A*) LocusZoom plot of Crohn's disease and ulcerative colitis associations at the *ERAP2* locus. (*y*-axis) −log_10_(*P*-value) of association; (*x*-axis) genomic location. (*B*) Structures of transcripts derived from each haplotype (*top*) and box-whisker plots (*bottom* three panels) between *ERAP2* transcript quantitative traits (*y*-axis: log_2_(normalized gene abundance), log_2_(normalized isoform abundance), or isoform usage percentage)) and genotype (*x*-axis). (*C*) Correlation between *ERAP2*/Iso1 (orange), *ERAP2*/Iso3 (light blue), and *ERAP2*/Iso4 (dark blue) abundance (*y*-axis) and abundance of flu segment 4 (*x*-axis) segregated by rs2248374 genotype (squares indicate aa; triangles, aG; circles, GG). (*D*) Western blot of MoDCs before and after flu-infection from five Haplotype B homozygotes and two heterozygotes. A full-length *ERAP2* protein isoform is expected at 120 kDa. At least one flu-specific *ERAP2* protein isoform is expected at 49 kDa. (*E*) A schematic of the hypothesized regulation and function of two *ERAP2* haplotypes. (N) N terminal domain; (AP) amino peptidase domain; (H) hinge domain; (C) C terminal domain; (PTC) premature termination codon.

The *ERAP2* locus is characterized by two frequent and highly differentiated (40 SNPs in perfect LD) haplotypes observed in every major human population (B: 53% and A: 47%) (Supplemental Fig. S15). The minor Haplotype A encodes a 965-amino-acid protein and is associated with Crohn's disease (Jostins et al. 2012) but not ulcerative colitis (Fig. 5A). The major allele (G) of rs2248374, a splice-site variant tagging Haplotype B, creates an alternate 3′ donor splice site inducing the alternative splicing of an extended exon 10 with two premature termination codons (Andres et al. 2010). As a result, transcripts from Haplotype B are degraded by nonsense-mediated decay, resulting in one of the most significant eQTLs and isoQTLs in most tissues and cell types (Lappalainen et al. 2013; Lee et al. 2014; Ye et al. 2014; The GTEx Consortium 2015). The *ERAP2* locus has been maintained by long-term balancing selection (between 1.4 million [Andres et al. 2010] and 5.1 million yr [Cagliani et al. 2010]), raising the important question: In what environmental conditions does balancing selection act to maintain the seemingly loss-of-function (LOF) Haplotype B and the disease-causing Haplotype A in humans?

Given the known role of *ERAP2* in antigen presentation (Saveanu et al. 2005), we examined the genetic control of *ERAP2* transcripts in the human antiviral response. In resting and IFNB1-stimulated cells, we confirmed the known genetic association of rs2248374^G^ allele with lower *ERAP2* expression (Fig. 5B). In flu-infected but not IFNB1-stimulated cells, two previously uncharacterized short isoforms (*ERAP2*/Iso3, *ERAP2*/Iso4) (Fig. 5B; Supplemental Fig. S16) were transcribed from Haplotype B, resulting in the partial rescue of *ERAP2* expression. The short isoforms differed from the constitutive full-length isoform (*ERAP2*/Iso1 transcribed from Haplotype A) by the initiation of transcription at exon 9 and the alternative splicing of an extended exon 10, and they differed from each other by alternative splicing at a secondary splice site at exon 15. The initiation of transcription at exon 9 results in an alternate in-frame translation start site at exon 11, thus rendering the premature termination codon in exon 10 inactive. The influenza-dependent genetic control of *ERAP2* isoform usage is supported by two additional lines of evidence. First, there was significant correlation between overall flu transcript abundance, a proxy for degree of infection, and *ERAP2*/Iso3 and *ERAP2*/Iso4 transcript abundances and usages in heterozygotes and Haplotype B homozygotes (Fig. 5C; Supplemental Fig. S17). Second, the transcription of an extended exon 10, a hallmark of flu-specific short isoforms, was observed in monocyte-derived macrophages infected by H3N2 over a time course in an independent RNA-seq data set (fluomics, GEO GSE97672) (Supplemental Fig. S18).

The predicted protein products of either *ERAP2* short isoforms would be missing the N-terminal (N) and aminopeptase (AP) domains. From *ERAP2*/Iso3, only one protein product is expected to be translated, which maintains a partial hinge (H) domain and the full alpha helical C-terminal (C) domain. Although *ERAP2*/Iso4 is predicted to harbor a premature termination codon that could lead to NMD, the presence of the transcript suggests the alternate translation of an isoform starting at exon 16 that maintains a partial alpha helical C-terminal domain. This calls into question whether the short *ERAP2* isoforms would function as an RNA or protein product. Western blotting with a full-length *ERAP2* antibody detects at least one short protein isoform (∼50 kDa) in flu-infected cells from Haplotype B homozygotes and heterozygotes, suggesting the translation of the short influenza-specific *ERAP2* isoforms (Fig. 5D; Supplemental Fig. S19).

We present a model of *ERAP2* regulation and function consistent with our findings and previous results (Fig. 5E). The genetic signals at the *ERAP2* locus suggest at least three perfectly linked variants on Haplotype B affecting *ERAP2* transcription and splicing in response to viral infection. Rs2548538, an intronic variant that overlaps chromatin marks from lymphoblastoid cell lines (The ENCODE Project Consortium 2012), is a candidate SNP that causes alternate transcript initiation at exon 9. Rs2248374^G^, the known splice site mutation, creates an alternate preferred splice site, resulting in alternative splicing of an extended exon 10. Rs2549797^G^, a splice-site mutation that creates a competing alternate splice site, results in ∼40% of the transcripts with an extended exon 15. Previous work has shown that full-length *ERAP2* is a prototypical aminopeptidase that homodimerizes and heterodimerizes with *ERAP1* (Saveanu et al. 2005) to perform the final peptide trimming step prior to MHC class I loading in the ER. The translation of the flu-specific *ERAP2* isoforms that lack the aminopeptidase domain suggests that it could act as a dominant negative to either *ERAP2* or *ERAP1* to disrupt normal antigen processing, creating a more immunogenic MHC peptide repertoire that could confer a fitness advantage in response to virus.

## Discussion

Although maps of genetic variants associated with overall gene abundances have been generated in many tissue types, the genetic control of alternate isoform usage has not been extensively studied. By using de novo transcript reconstruction, we found a large number of previously uncharacterized transcripts in human MoDCs, especially in response to influenza and interferon stimulation, indicating that the current reference human transcriptome is far from complete. However, de novo reconstruction using short reads has fundamental limitations especially for longer genes with multiple splice junctions (Steijger et al. 2013). A hybrid approach that uses long-read sequencing (Byrne et al. 2017; Tardaguila et al. 2018) to scaffold and short-read sequencing to assemble and quantify transcripts should result in higher-quality reference transcriptomes.

We found that genetic variants (isoQTLs) associated with alternate isoform usage are widespread, >40% of which are not associated with the overall abundance of the corresponding gene, indicative of independent genetic control of gene regulation at these loci. The enrichments of isoQTLs for known splice sites and autoimmune and neurodegenerative disease loci suggest a highly clinically relevant set of candidate variants that induce changes in protein sequence. To further assess the landscape of genetic control of isoform usage across immune cell types and stimuli, bulk sequencing in sorted cell populations or cost-effective single-cell sequencing (Kang et al. 2018) could be performed.

To conclusively establish the consequences of isoQTLs on protein function would require profiling of protein isoforms in large population cohorts, which remains a challenging task. Indeed, other studies have shown that much of the genetic effects on overall transcript abundance has little effect on overall protein abundance, likely due to buffering by post-transcriptional processes (Li et al. 2016). One independent approach to assess isoform function would be to compare transcript structure and abundances across species. Although several studies have begun to undertake this task across mammals for a number of tissues (Barbosa-Morais et al. 2012; Merkin et al. 2012), studies in immune cell types in response to specific stimuli are still lacking.

IsoQTLs, like eQTLs, can affect gene expression at distal loci in the genome, suggesting important downstream effects on gene regulation. The most striking example is at the *IRF7* locus, where a splice-site SNP affects *IRF7* splicing in response to influenza and interferon but only affects the expression of downstream genes in response to flu. This suggests that both genetic effects on isoform usage and stimulation-dependent regulation of *IRF7* expression are necessary for the observed *trans* effects. Although C-terminal splice forms of *IRF7* have been shown to differentially transactivate type 1 interferons and chemokines (Lin et al. 2000), *IRF7*/Iso4 is not known to have specific antiviral properties in vivo even though its ectopic expression is known to activate IFNAs in fibroblasts (Au et al. 1998). The association of the variant with SLE indicates a possible role for viral exposure to prime the immune system of individuals carrying the risk allele toward autoimmunity.

Genetic variants at a single locus could affect multiple facets of gene regulation in response to stimulation to establish variability in transcript structure and abundance. This was clearly demonstrated at the *ERAP2* locus, where multiple variants result in differential expression and splicing of short isoforms in response to influenza but not interferon. The lack of expression in response to IFNB1 suggests the transcription of novel *ERAP2* isoforms is likely initiated by viral sensing pathways upstream of type 1 interferon signaling. Furthermore, balancing selection at *ERAP2* suggests that transcripts derived from both haplotypes could confer fitness advantages, likely in different environments. We provide evidence of the expression of short *ERAP2* isoforms encoded by Haplotype B in response to influenza, suggesting viral infection as a possible selective agent. One can speculate that the long *ERAP2* isoform encoded by Haplotype A could be selected under different environmental conditions that favor an overactive autoinflammatory response or a primed T-cell response. Altogether, the genetic analysis of isoforms under physiologically relevant conditions can help reveal new gene regulatory mechanisms by which alleles associated with disease and under natural selection function in response to environment.

## Methods

### Study subjects and sample preparation

Donors were recruited from the Boston community and gave written informed consent for the studies. Individuals were excluded if they had a history of inflammatory disease, autoimmune disease, chronic metabolic disorders, or chronic infectious disorders. Donors were between 18 and 56 yr of age (average 29.9). As previously described (Lee et al. 2014), 35–50 mL of peripheral blood from fasting subjects was collected between 7:30 and 8:30 am. The blood was drawn into sodium heparin tubes, and peripheral blood mononuclear cells (PBMCs) were isolated by Ficoll-Paque (GE Healthcare Life Sciences) centrifugation. PBMCs were frozen in liquid N_2_ in 90% FBS (Sigma-Aldrich) and 10% DMSO (Sigma-Aldrich). Monocytes were isolated from PBMCs by negative selection using the Dynabeads untouched human monocytes kit (Life Technologies) modified to increase throughput and optimize recovery and purity of CD14^+^CD16^lo^ monocytes: The FcR blocking reagent was replaced with Miltenyi FcR blocking reagent (Miltenyi); per milliliter of antibody mix, an additional 333 µg biotinylated anti-CD16 (Biolegend, catalog no. 302004), 167 µg biotinylated anti-CD3 (Biolegend, catalog no. 344820), and 167 µg biotinylated anti-CD19 (Biolegend, catalog no. 302204) antibodies were added; the antibody labeling was modified to be performed in 96-well plates; and Miltenyi MS columns or multi-96 columns (Miltenyi) were used to separate magnetically labeled cells from unlabeled cells in an OctoMACS separator or MultiMACS M96 separator (Miltenyi), respectively. The number of PBMCs and monocytes was estimated using a CellTiter-Glo luminescent cell viability assay (Promega). A subset of the isolated monocytes was stained with PE-labeled anti-CD14 (M5E2; BD Biosciences) and FITC-labeled anti-CD16 (3G8; Biolegend) and was subjected to flow cytometry analysis using an Accuri C6 flow cytometer (BD Biosciences). A median of 94% CD14^+^ cells and 99% CD16^lo^ cells was obtained.

### Differentiation and stimulation of primary human MoDCs

Monocytes were cultured for 7 d in RPMI (Life Technologies) supplemented with 10% FBS, 100 ng/mL GM-CSF (R&D Systems), and 40 ng/mL IL-4 (R&D Systems) to differentiate the monocytes into MoDCs; 4 × 10^4^ MoDCs were seeded in each well of a 96-well plate. Cells were left unstimulated or were stimulated either with Influenza A (PR8 ΔNS1, prepared as previously described) (Shapira et al. 2009) for 10 h or with 100 U/mL recombinant IFNB1 (PBL Assay Science) for 6.5 h. Cells were then lysed in RLT buffer (Qiagen) supplemented with 1% B1-mercaptoethanol (Sigma-Aldrich).

### RNA isolation and sequencing

RNA from all samples was extracted using the RNeasy 96 kit (Qiagen, catalog no. 74182), according to the manufacturer's protocols. Five hundred seventy-six total samples were sequenced (99 baseline, 250 influenza infected, and 227 IFNB1 stimulated). Five hundred fifty-two pass-filter samples (94 baseline, 243 influenza, and 215 interferon) were sequenced to an average depth of 38 million 76-bp paired-end reads using the Illumina TruSeq kit. Samples were filtered if the genotypes estimated from the RNA-sequencing did not match the genotypes obtained from genotyping (19 samples) or if self-reported ethnicity did not match the ethnicity estimated from genotypes (five samples) (Supplemental Table S1). Reads were aligned to hg19 genome with TopHat v1.4.1 (--mate-inner-dist 300 --mate-std-dev 500) (Trapnell et al. 2009) with 86% mapping to transcriptome and 97% mapping to the genome (Supplemental Table S1). Realigning our data to hg38 (GRCh38) would not significantly affect our conclusions: hg38 (GRCh38) primarily improves from hg19 (GRCh37) in centromeric and other repetitive regions in the human genome that were not included in our analysis due to ambiguity from mapping short RNA-seq reads. It is estimated that hg38 increases the number of mappable mRNAs by 3% (Schneider et al. 2017) and thus minimally impacts our RNA-seq–based study. Further, as all our samples were mapped to hg19, any sequence errors that introduced artifacts into read mapping would have affected all the samples equally and would not have yielded significant differential expression or splicing results.

### Transcriptome reconstruction

After aligning reads to the genome, transcriptomes were assembled for each sample individually using StringTie (Pertea et al. 2015) and default parameters. Abundances of annotated transcripts were quantified using Kallisto (Bray et al. 2016). For genes expressed at more than five TPM in any sample, isoforms expressed at less than five TPM across all samples were removed. In order to reduce the number of transfrags, transcriptomes across the same condition (e.g., baseline, flu, IFNB1) were first merged using cuffmerge (--overhang-tolerance 0) (Trapnell et al. 2012). Merged transcriptomes across all three conditions were then combined, and redundant isoforms were removed using cuffcompare (Trapnell et al. 2012).

### Transcriptome comparison

For each reconstructed isoform, the position of each splice site, as well as the 5′ and 3′ position of each splice junction, was compared with annotated isoforms from GENCODE (v27) (Harrow et al. 2006), UCSC (hg19) (Casper et al. 2018), and RefSeq (hg19) (O'Leary et al. 2016), and comparison statistics for the most similar annotated isoform were reported. To quantify the coverage of spliced reads across splice junctions, each TopHat alignment was inputted into LeafCutter (Li et al. 2018), which uses the CIGAR strings in alignment BAM files to count the number of high-quality aligning reads at each splice junction. To detect novel TSSs, we checked the first 100 bp of our reconstructed isoforms for overlap with TSSs of annotated isoforms. Novel TSSs detected in our data set were further compared with CAGE reads from MoDCs from the FANTOM5 database (FF:11227-116C3, F:11308-117C3, FF:11384-118B7) (Noguchi et al. 2017) in the following way: First, a specialized transcriptome consisting of only the first 500 bp of each de novo assembled transcript (stranded, and after splicing) was created. Then, CAGE reads were aligned to this specialized transcriptome using Bowtie v0.12.7 with default parameters (Langmead 2010). Finally, the presence of each TSS was quantified by counting the number of mapped reads.

### Differential expression analysis

Isoform level differential expression testing was carried out with sleuth (Pimentel et al. 2017) using 100 bootstraps per sample. Gene-level quantification was estimated by summing isoform counts from Kallisto, and differential expression testing was carried out with DESeq2 (Anders and Huber 2010).

### Differential isoform usage analysis

Differential isoform usage testing was carried out in R using the beta regression package betareg (Cribari-Neto and Zeileis 2010), and *P*-values were calculated using a likelihood ratio test and adjusted with a false-discovery rate adjustment.

### Gene Ontology enrichment analysis

Gene Ontology (GO) enrichment analysis was carried out using GOrilla (Eden et al. 2009) and tested against a background of only the set of genes that were expressed in MoDCs and that we recovered during the transcriptome reconstruction (see above).

### DNA extraction and genotyping

As previously described (Lee et al. 2014), genomic DNA was extracted from 5 mL whole blood (DNeasy blood & tissue kit; Qiagen) and quantified by NanoDrop. Each subject was genotyped using Illumina infinium human OmniExpress exome BeadChip, which includes genome-wide genotype data as well as genotypes for rare variants from 12,000 exomes as well as common coding variants from the whole genome. In total, 951,117 SNPs were genotyped, of which 704,808 SNPs are common variants (minor allele frequency [MAF] > 0.01) and 246,229 are part of the exomes. The genotype success rate was ≥97%. We applied rigorous subject and SNP quality control (QC) that includes (1) gender misidentification, (2) subject relatedness, (3) Hardy-Weinberg equilibrium testing, (4) use concordance to infer SNP quality, (5) genotype call rate, (6) heterozygosity outlier, and (7) subject mismatches. In the European population, we excluded 1987 SNPs with a call rate <95%, 459 SNPs with Hardy-Weinberg equilibrium *P*-value <10^−6^, 234 SNPs with a MisHap *P*-value <10^−9^, and 63,781 SNPs with MAF < 1% from a total of 66,461 SNPs excluded. In the African-American population, we excluded 2161 SNPs with a call rate <95%, 298 SNPs with Hardy-Weinberg equilibrium *P*-value <10^−6^, 50 SNPs with a MisHap *P*-value <10^−9^, and 17,927 SNPs with MAF <1% from a total of 20,436 SNPs excluded. In the East Asian population, we excluded 1831 SNPs with a call rate <95%, 213 SNPs with Hardy-Weinberg equilibrium *P*-value <10^−6^, 47 SNPs with a MisHap *P*-value <10^−9^, and 84,973 SNPs with MAF <1% from a total of 87,064 SNPs excluded. After QC, approximately 18,000–88,000 SNPs in each population were filtered out from our analysis. Underlying genetic stratification in the population was assessed by multidimensional scaling using data from The International HapMap Project (The International HapMap Consortium 2003) (CEU, YRI and CHB samples) combined with IBS cluster analysis using EIGENSTRAT 3.0 software (Price et al. 2006). The QC of the genotyping data was performed using PLINK (Purcell et al. 2007).

### Genotype imputation

To accurately evaluate the evidence of association signal at variants that are not directly genotyped, we used BEAGLE (Browning and Browning 2016) software (v3.3.2) to impute the post-QC genotyped markers using reference haplotype panels from The 1000 Genomes Project (The 1000 Genomes Project Consortium Phase I Integrated Release Version 3) (Siva 2008), which contain a total of 37.9 million SNPs in 1092 individuals with ancestry from West Africa, East Asia, and Europe. For subjects of European and East Asian ancestry, we used haplotypes from Utah residents (CEPH) with Northern and Western European ancestry (CEU) and combined panels from Han Chinese in Beijing (CHB) and Japanese in Tokyo (JPT), respectively. For imputing African American subjects, we used a combined haplotype reference panel consisting of CEU and Yoruba in Ibadan, Nigeria (YRI). For the admixed African American population, using two reference panels substantially improves imputation performance. After genotype imputation, we filtered out poorly imputed (BEAGLE *r*^2^ < 0.1) and low MAF SNPs (MAF < 0.01), which resulted in 7.7 million, 6.6 million, and 12.7 million common variants in European, East Asian, and African American, respectively. This set of genotyped and imputed markers was used for all the subsequent association analysis.

### Local eQTL and isoQTL mapping

QTL mapping was performed using the Matrix eQTL (Shabalin 2012) package using empirically determined number of principal components (PCs) as covariates for each analysis. The isoform usage ratio or log of normalized total gene abundance was regressed against all genetics variants with a MAF >5% in a 1-Mb (±500 kb) window, and the most significant association is kept. Zero to 44 PCs (local eQTLs) and zero to 12 PCs (local isoQTLs) in increments of two were tested, and the number of PCs was chosen to maximize the number of local e/isoQTLs detected (Supplemental Figs. S2, S3). Because of the smaller number of individuals in the baseline condition, the number of PCs adjusted was fewer (Supplemental Fig. S2). Because the isoform usage percentage implicitly adjusts for confounders that affect overall gene abundance and isoform abundance levels (i.e., other eQTLs), the number of adjusted PCs was also fewer (Supplemental Fig. S3). Experiment-wide empirical *P*-values were calculated by comparing the nominal *P*-values with null *P*-values determined by permuting each isoform/gene 1000 times (Churchill and Doerge 1994). The permutation *P*-values were not pooled to calculate the empirical *P*-values (i.e., the minimum possible *P*-value is 0.001). False-discovery rates were calculated using the qvalue package (https://github.com/StoreyLab/qvalue) as previously described (Storey and Tibshirani 2003).

### Independence of eQTLs and isoQTLs

We examined the overlap between eQTLs and isoQTLs to better understand the underlying mechanisms by which differential isoform usage is achieved. There could be two genetic architectures that result in an isoQTL. Suppose we have two transcripts *A*_1_ and *A*_2_ for gene *A*. Consider the first architecture, SNP *G*_1_, affects the transcription of *A*_1_ but not *A*_2_: $A_{1}∼NB(\mu_{1}=\mu_{0}+G_{1}\times \beta ,r=\mu_{1}/3)$ and $A_{2}∼NB(\mu_{2}=\mu_{0},r=\mu_{2}/3)$. Compared with using log(transcript *A*_1_ abundance) as a trait, detecting an eQTL by fitting linear regression using log(total abundance) and detecting an isoQTL using isoform usage ratio would both have reduced power (Supplemental Figure S6). Consider the second architecture, SNP *G*_2_, affects the splicing of *A*_1_ versus *A*_2_: $A_{1}∼NB(\mu_{1}=\mu_{0}+G_{2}\times \beta ,r=\mu_{1}/3)$ and $A_{2}∼NB(\mu_{1}=\mu_{0}-G_{2}\times \beta ,r=\mu_{1}/3)$. Compared with using log(transcript *A*_1_ abundance) as a trait, detecting an eQTL by fitting linear regression using log(total abundance) would have no power, and detecting an isoQTL using isoform usage ratio would have increased power (Supplemental Figure S7). We obtained similar results by performing negative binomial regression. The parameters used for the simulation were *N* = 100, *MAF* = 0.5, *μ*_0_ = 500. The size parameter for the negative binomial was chosen based on published recommendations (Frazee et al. 2015).

### QTL annotation

QTLs were annotated using Variant Effect Predictor and Ensembl release 79 (McLaren et al. 2016). Exonic and intronic locations of QTLs were determined using UCSC's canonical transcripts (table *knownCanonical*) as a reference (Karolchik et al. 2004). Enrichments were calculated against background set of SNPs that were matched in allele frequency (binned by 4%) and distance to nearest TSS (binned by 10 kb).

### Overlap with GWAS associations

The GREGOR suite (Schmidt et al. 2015) was used for calculating the enrichment of eQTLs and isoQTLs containing a GWAS loci across baseline, flu, and IFNB1 stimulations. GWAS associations for disease with FDR < 0.1 were reported.

### Partitioned heritability analysis

We used ldsc with default parameters, which implements LD score regression (Finucane et al. 2015) to calculate the proportion of SNP heritability explained by eQTL/isoQTLs. We obtained summary statistics from 28 human traits/diseases from https://data.broadinstitute.org/alkesgroup/sumstats_formatted/.

### Estimating flu transcript abundance

Flu transcript abundance was estimated by using RSEM (Li and Dewey 2011) to map RNA-seq data to a custom reference of the influenza PR8 genome (Supplemental Material).

### ERAP2 western blot

Protein extracts were fractionated by SDS-PAGE (4%–12% Bis-Tris gel, Thermo Fisher Scientific, NP0335BOX) and transferred to PVDF membrane (Bio-Rad, catalog no. 162-0177). After blocking with 2% BSA in TBST (Tris buffered saline containing 0.1% Tween 20) for 1 h, membranes were incubated with primary antibody (either ERAP2, R&D Systems, catalog no. AF3830, 1:3000) or actin beta (Abcam, catalog no. ab6276, 1:15,000) overnight at 4°C. Membranes were then washed and incubated with a 1:5000 dilution of HRP conjugated secondary antibody (either donkey anti-goat from Santa Cruz Biotech, catalog no. sc2020, or with goat anti-mouse from Jackson Immune Research, catalog no. 115-035-146) for 1 h. Membranes were washed and developed with ECL system (VWR, catalog no. 89168-782) according to the manufacturer's protocol.

## Data access

RNA-seq raw data from this study have been submitted to the NCBI database of Genotypes and Phenotypes (dbGaP; https://www.ncbi.nlm.nih.gov/gap) under accession number phs000815.v1.p1. RNA-seq processed data from this study have been submitted to the NCBI Gene Expression Omnibus (GEO; http://www.ncbi.nlm.nih.gov/geo/) under accession number GSE92904.

## Competing interest statement

N.H. is a founder and SAB member of Neon Therapeutics and a SAB member of IFM Therapeutics. A.R. is a SAB member of Thermo Fisher Scientific, Syros Pharmaceuticals, and Driver group and a founder of Celsius Therapeutics.

## Supplementary Material

Supplemental Material
