# Supplementary material for: Genetic analysis of isoform usage in the human anti-viral response reveals influenza-specific regulation of ERAP2 transcripts under balancing selection
Source: Genome Res. 2018 Dec;28(12):1812–25. doi: 10.1101/gr.240390.118 (PMC6280757; doi:10.1101/gr.240390.118)
Supplement: Supplemental Material [file supp_28_12_1812__index.html]

Genetic analysis of isoform usage in the human anti-viral response reveals influenza-specific regulation of ERAP2 transcripts under balancing selection — Supplemental Material 

# Genetic analysis of isoform usage in the human anti-viral response reveals influenza-specific regulation of *ERAP2* transcripts under balancing selection

## Supplemental Material

- SupplementalTable6.xlsx
- SupplementalTable7.xlsx
- SupplementalTable8.xlsx
- Supplemental\_Materials.docx
- SupplementalTable3.xlsx
- SupplementalTable1.xlsx
- SupplementalTable4.xlsx
- SupplementalTable2.xlsx
- SupplementalTable5.xlsx
