# Supplementary material for: Genetic analysis of isoform usage in the human anti-viral response reveals influenza-specific regulation of ERAP2 transcripts under balancing selection
Source: Genome Res. 2018 Dec;28(12):1812–25. doi: 10.1101/gr.240390.118 (PMC6280757; doi:10.1101/gr.240390.118)
Supplement: Supplemental Material [file supp_gr.240390.118_Supplemental_Materials.docx]

**Supplementary Figures**

**Fig. S1** – Histogram of log_10_(TPM) of genes with unannotated isoforms. By manual inspection, we chose expression cutoff of 25 TPM (dotted line) for filtering out genes with low quality isoform reconstructions.

**
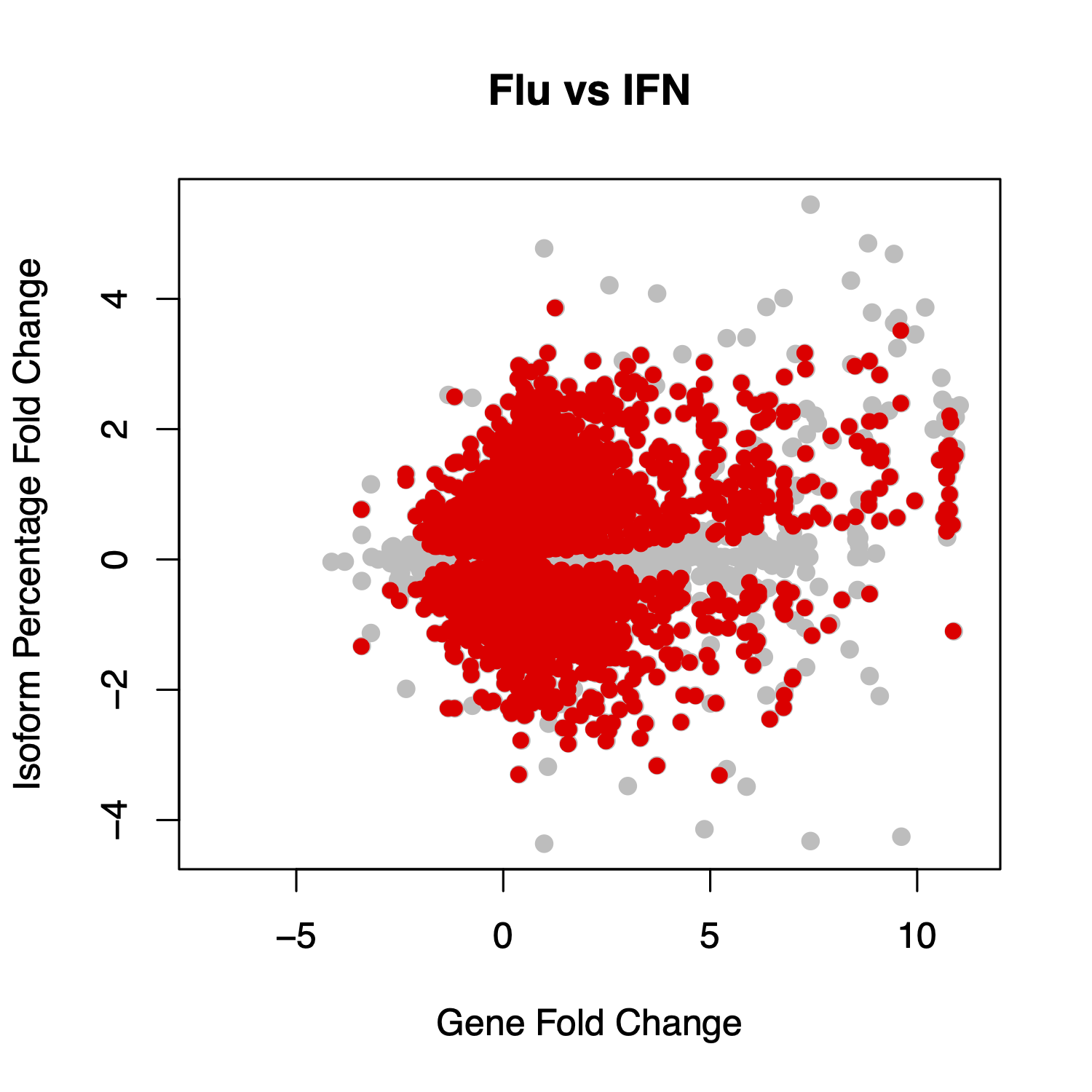
**

**Fig. S2** – Scatter plot of log_2_ fold change of overall gene abundance (x-axis) versus log_2_ fold change in isoform percentage (y-axis) in flu-infected cells compared to IFNB1-simulated cells. Each dot represents one isoform. Isoforms that significantly differed in their usage percentages (beta regression, FDR < 0.05) are highlighted in red.


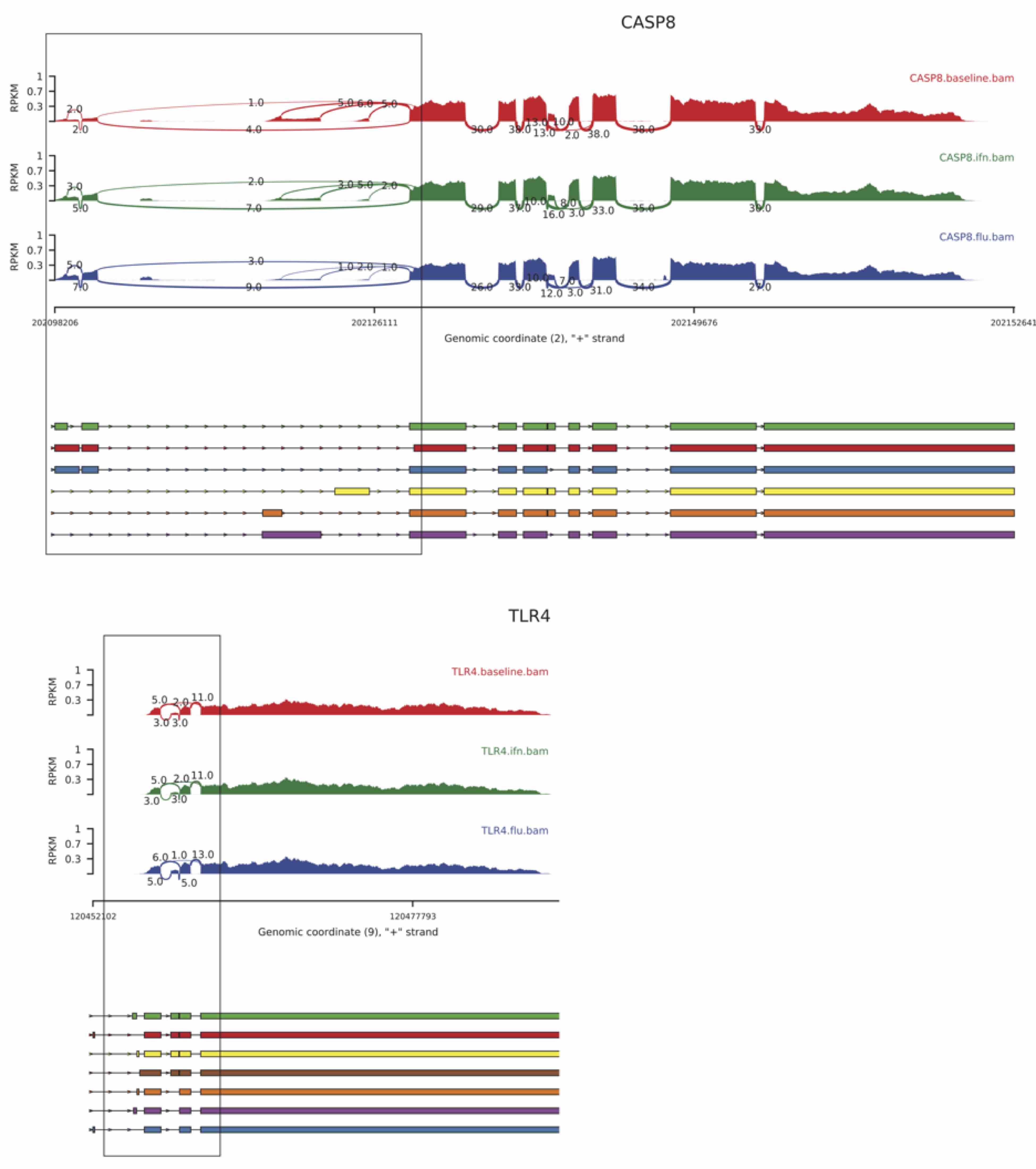


**Fig. S3** – Sashimi plots (top) and gene structure (bottom) of *CASP8* and *TLR4* junctions in response to stimulation.

**Fig. S4** – Empirically determined number of principal components (PCs) to adjust for eQTLs.

**Fig. S5** – Empirically determined number of PCs to adjust for isoQTLs.


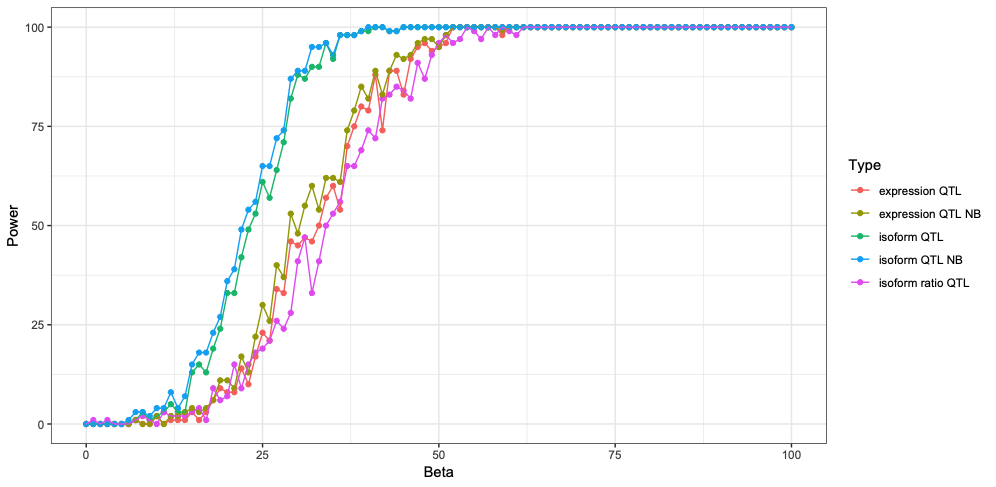


**Fig. S6** – Power (y-axis) to detect a QTL for a range of betas (x-axis) and $N=100$, assuming $MAF=0.5$ and the following genetic architecture: SNP $G_{1}$ affects the transcription of $A_{1}$ but not $A_{2}$: $A_{1}\sim NB\left( \mu_{1}=\mu_{0}+G_{1}\times\beta,r=\mu_{1}/3 \right)$ and $A_{2}\sim NB\left( \mu_{2}=\mu_{0},r=\mu_{2}/3 \right)$. Results from simulations based on both normally distributed and negative binomial (NB) data are shown.


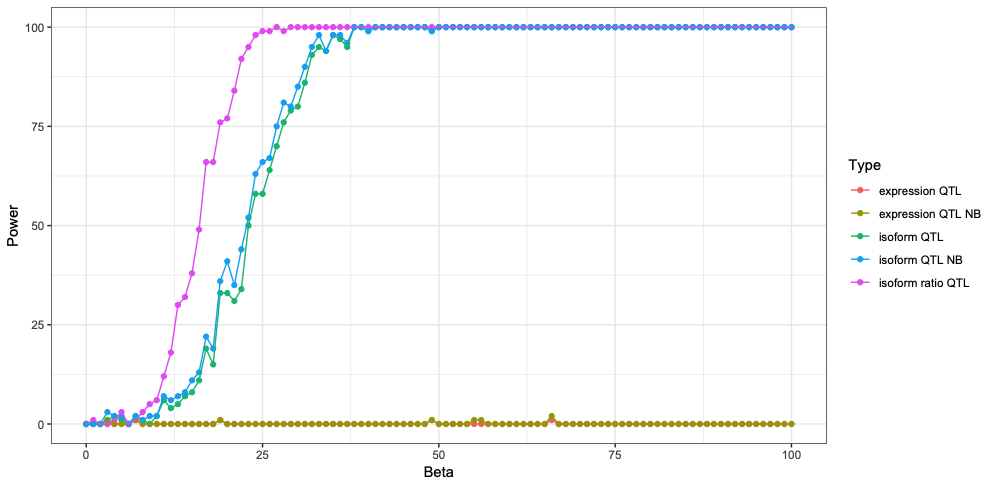
**Fig. S7** – Power (y-axis) to detect a QTL for a range of betas (x-axis) and $N=100$, assuming $MAF=0.5$ and the following genetic architecture: SNP $G_{2}$ affects the splicing of $A_{1}$ vs $A_{2}$: $A_{1}\sim NB\left( \mu_{1}=\mu_{0}+G_{2}\times\beta,r=\mu_{1}/3 \right)$ and $A_{2}\sim NB\left( \mu_{1}=\mu_{0}-G_{2}\times\beta,r=\mu_{1}/3 \right)$. Results from simulations based on both normally distributed and negative binomial (NB) data are shown.


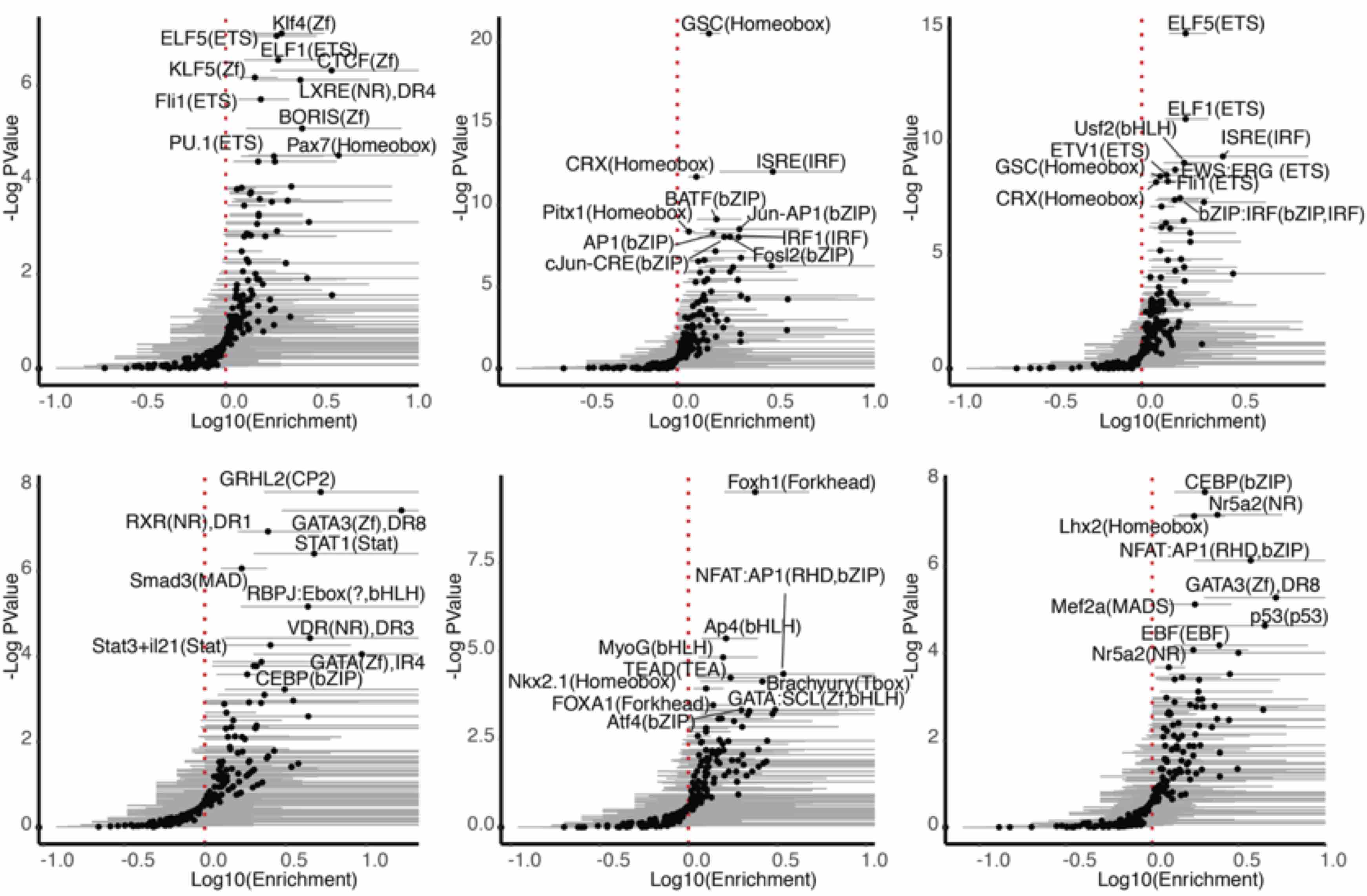


**Fig. S8** – Enrichment of eQTLs (top 3 panels) and isoQTLs (bottom 3 panels) for known transcription factor binding sites.


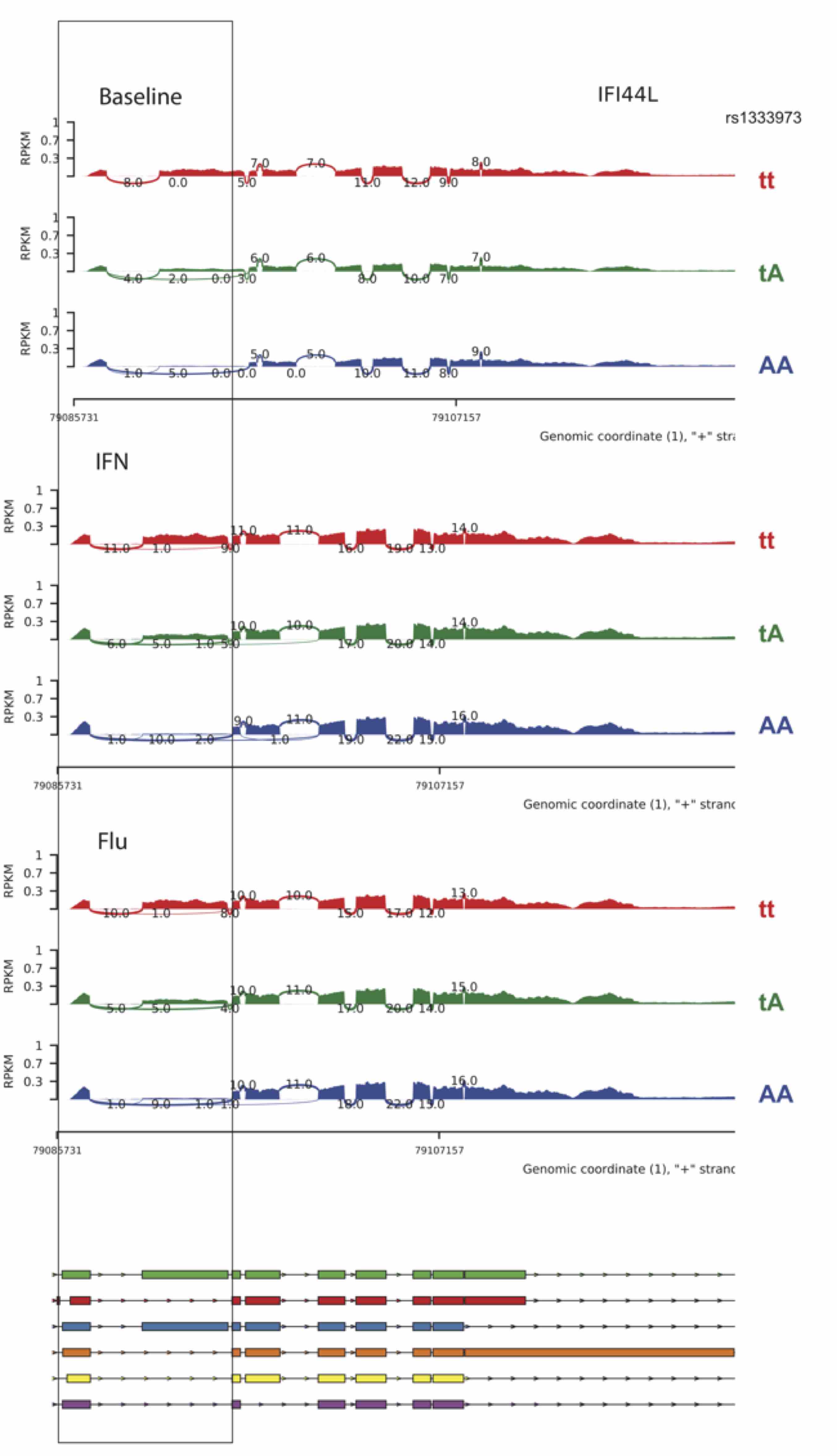


**Fig. S9** – Sashimi plot of *IFI44L* expression at baseline, or in response to interferon or influenza as a function of patient genotype at rs1333973. Key junctions are highlighted.


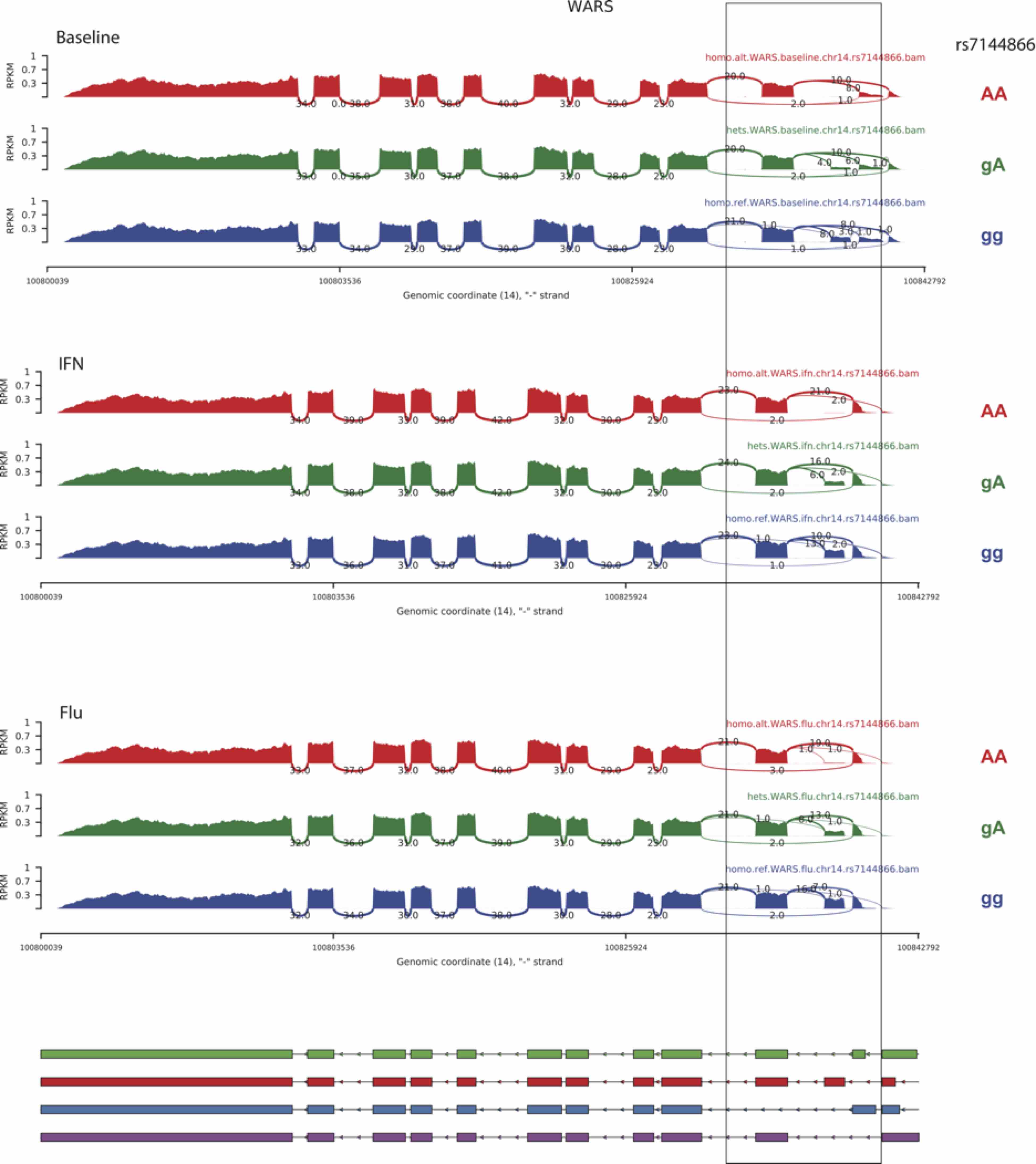


**Fig. S10** – Sashimi plot of *WARS* expression at baseline, or in response to interferon or influenza as a function of patient genotype at rs7144866. Key junctions are highlighted.


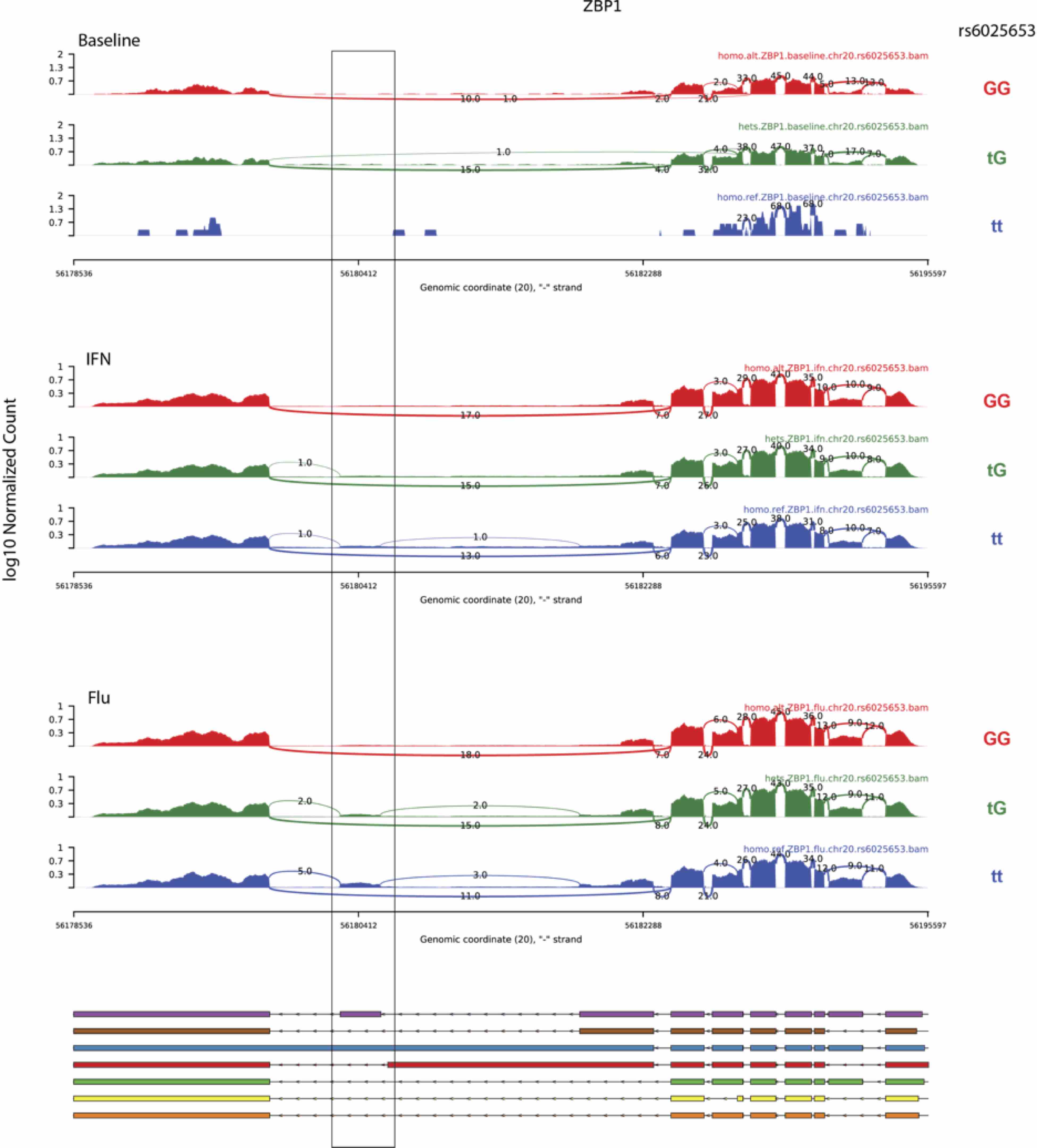


**Fig. S11** – Sashimi plot of *ZBP1* expression at baseline, or in response to interferon or influenza as a function of patient genotype at rs6025653. Key junctions are highlighted.


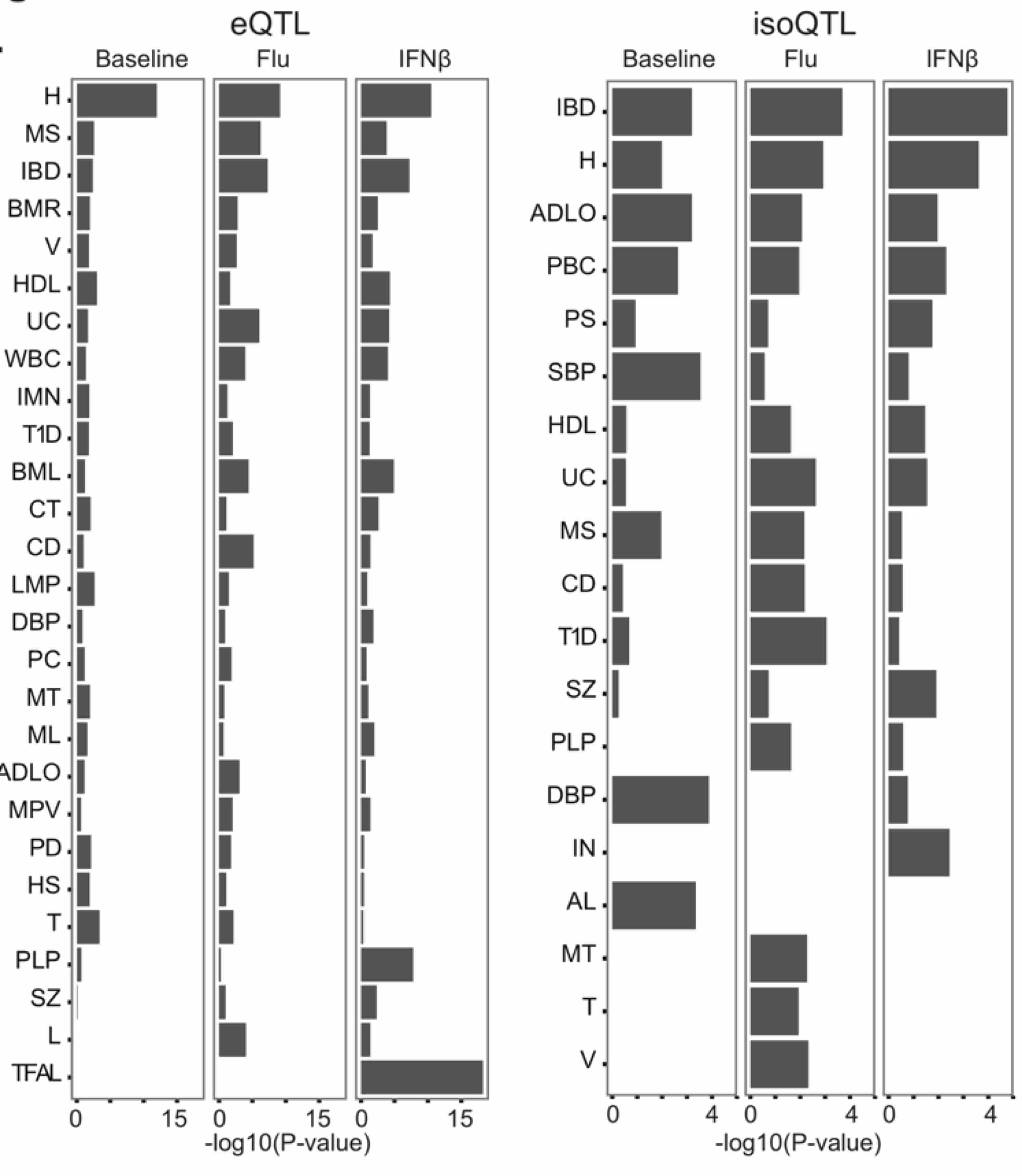


**Fig. S12**. Enrichment of leading eQTL and isoQTL SNPs for GWAS hits. Only diseases at a FDR < 0.1 for significant local e/isoQTLs (permutation FDR < 0.05) detected at baseline, post flu-infection or IFNB1-stimulation are plotted. X-axis: -log_10_(hypergeometric enrichment P-value). H: Height, MS: Multiple sclerosis, IBD: inflammatory bowel disease, BMR: basal metabolic rate, V: vertiligo, HDL: high-density lipoprotein, LDL: low-density lipoprotein, UC: ulcerative colitis, WBC: white blood count, IMN: idiopathic membranous nephropathy, T1D: type-1 diabetes, BML: blood metabolite levels, CT: cholesterol total, CD: Crohn’s disease, LMP: lipid metabolism phenotypes, DBP: diastolic blood pressure, PC: platelet counts, MT: metabolic traits, ML: metabolite levels, ADLO: Alzheimer’s disease late onset, MPV: mean platelet volume, PD: Parkinson’s disease, HS: hypospadias, T: triglycerides, PLP: phospholipid levels plasma, SZ: schizophrenia, L: leprosy, TFAL: trans fatty acid levels, PBC: primary biliary cirrhosis, PS: psoriasis, SBP: systolic blood pressure, DBP: diastolic blood pressure, IN: IgA nephropathy, AL: adiponectin levels.


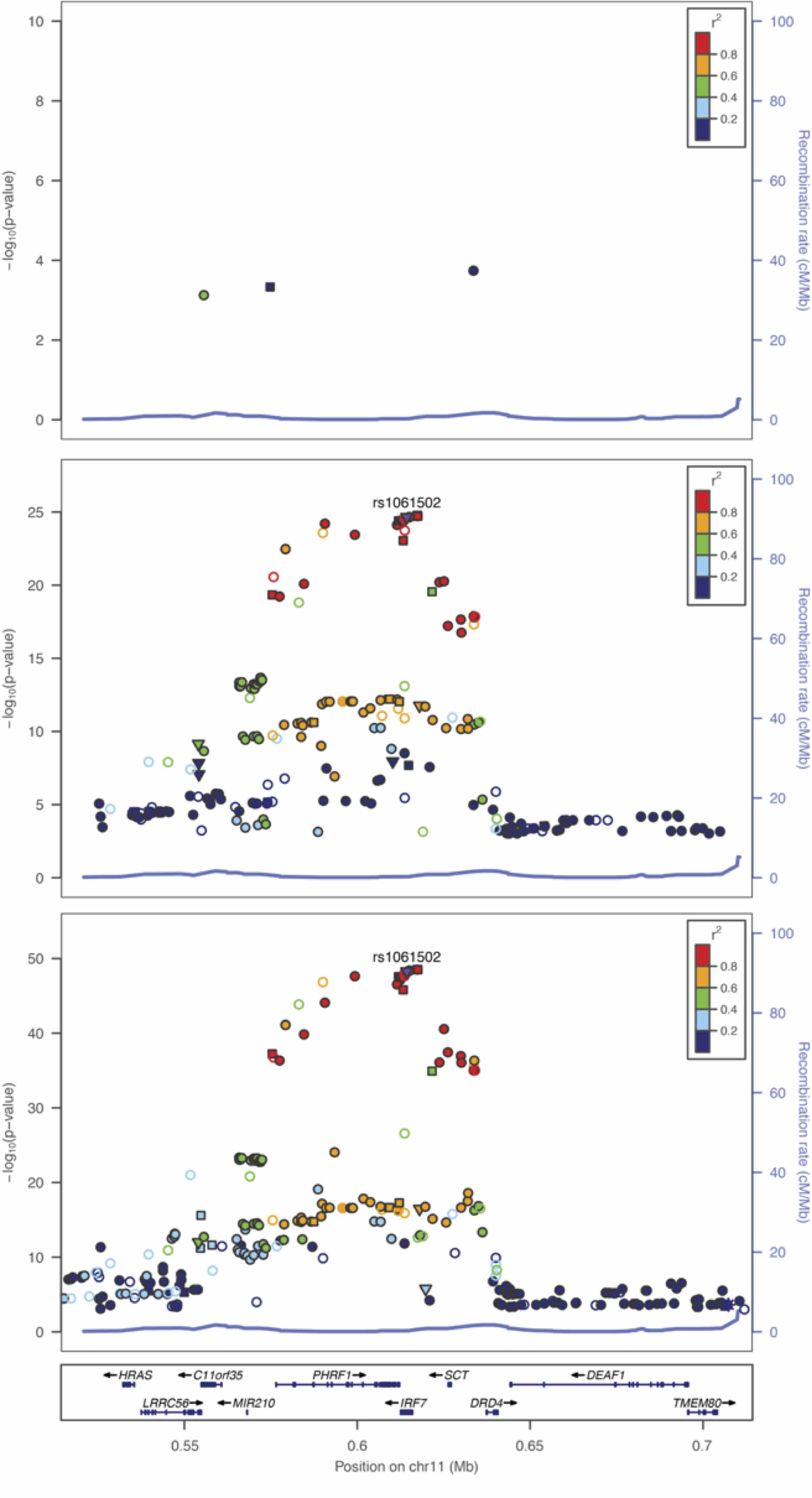


**Fig. S13** – LocusZoom plot for *IRF7* eQTLs.


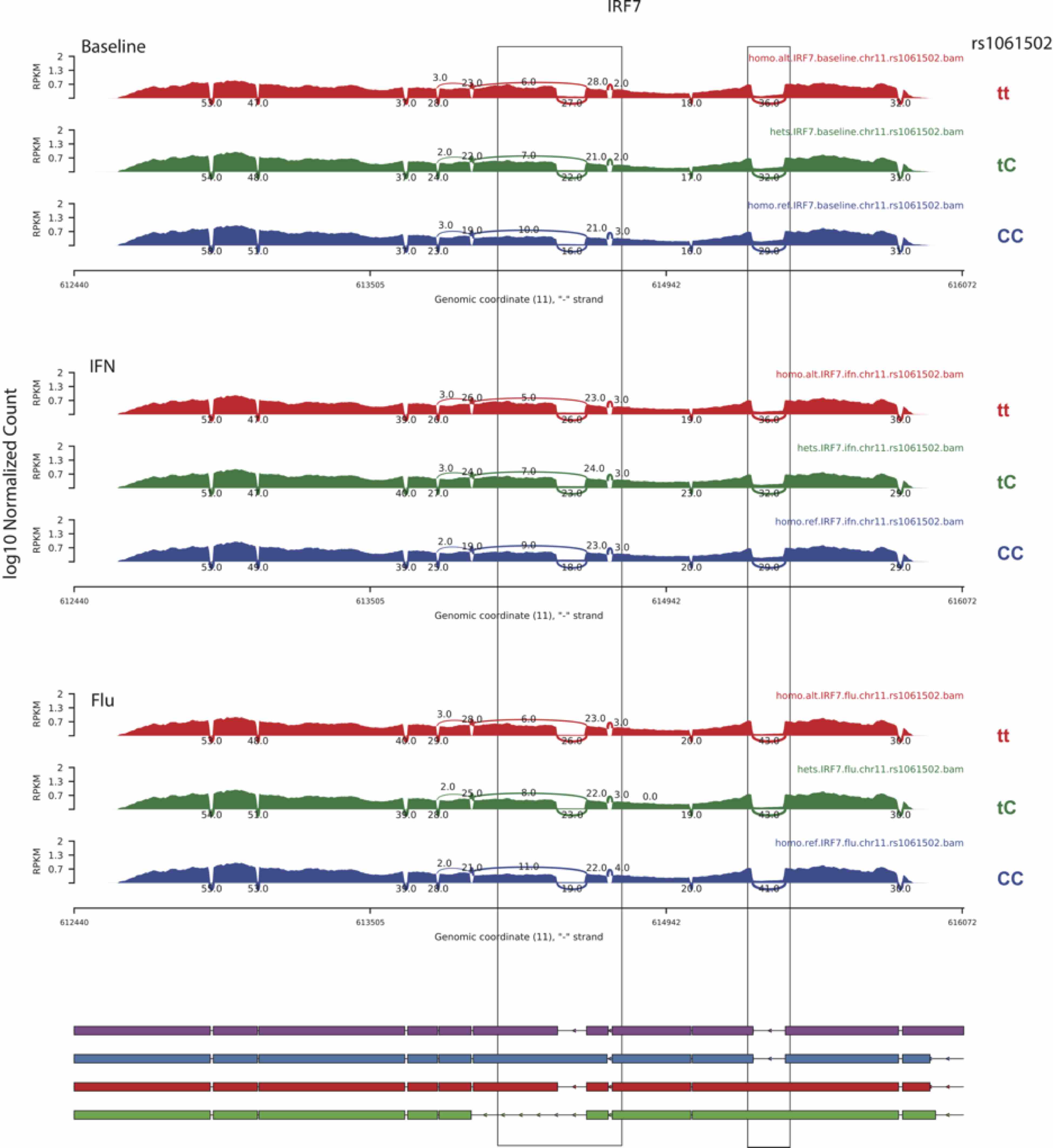


**Fig. S14** – Sashimi plot of *IRF7* expression at baseline, or in response to interferon or influenza as a function of patient genotype at rs1061502. Key junctions are highlighted.


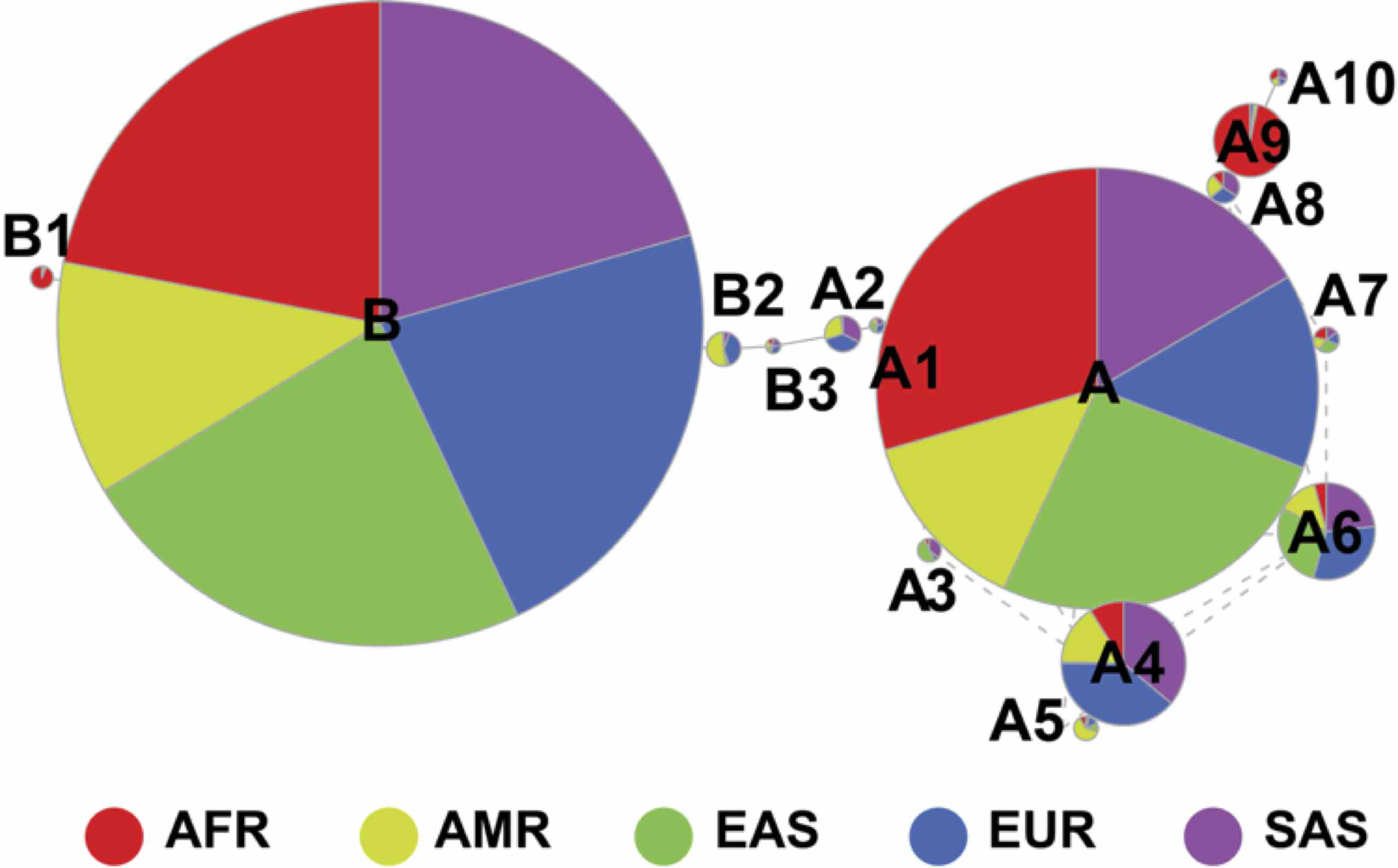


**Fig. S15** - Distribution of *ERAP2* haplotypes based on 1000 Genomes.


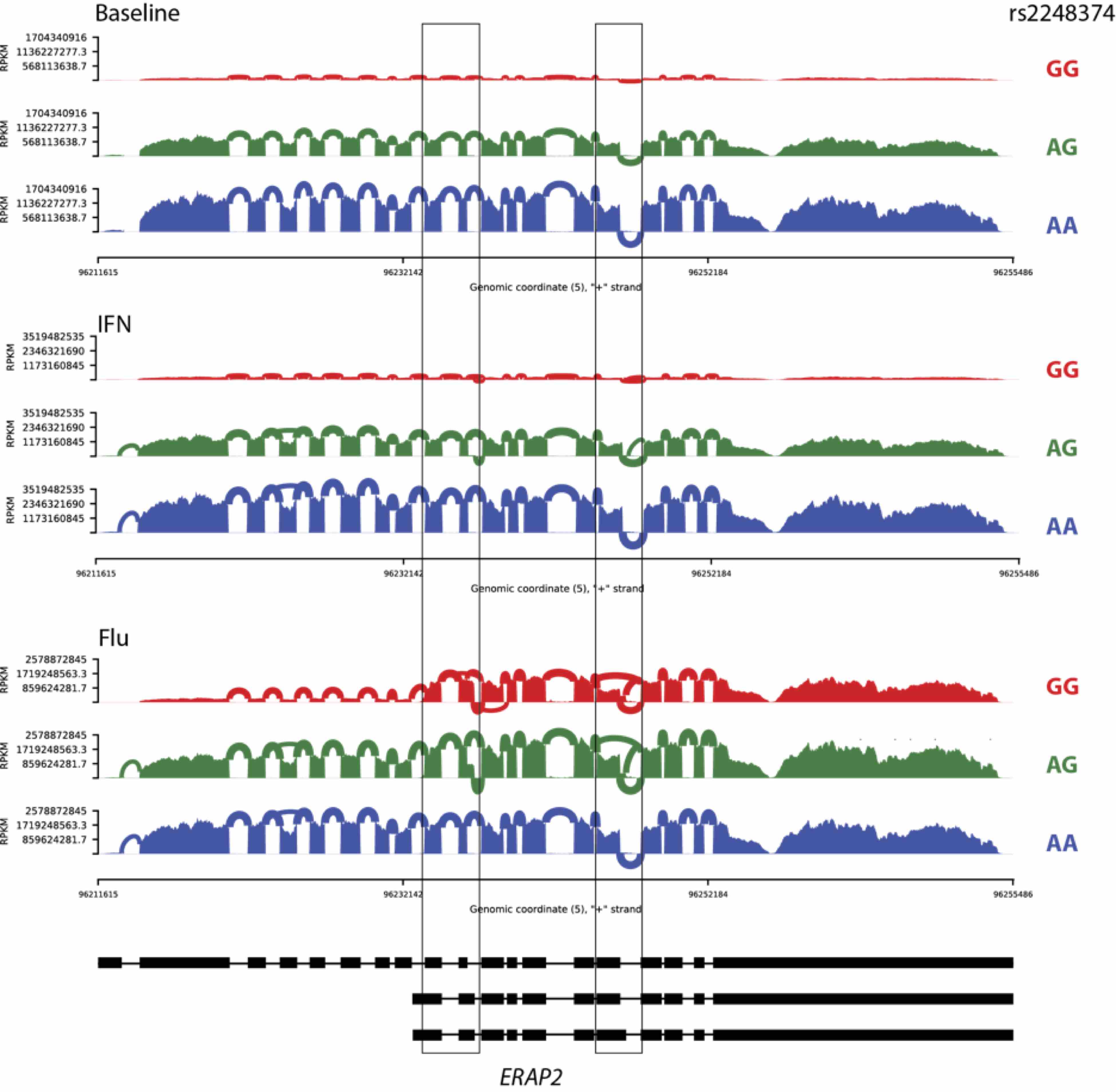


**Fig. S16** – Sashimi plot of *ERAP2* expression at baseline, or in response to interferon or influenza as a function of patient genotype at rs2248374. Key junctions are highlighted.

**
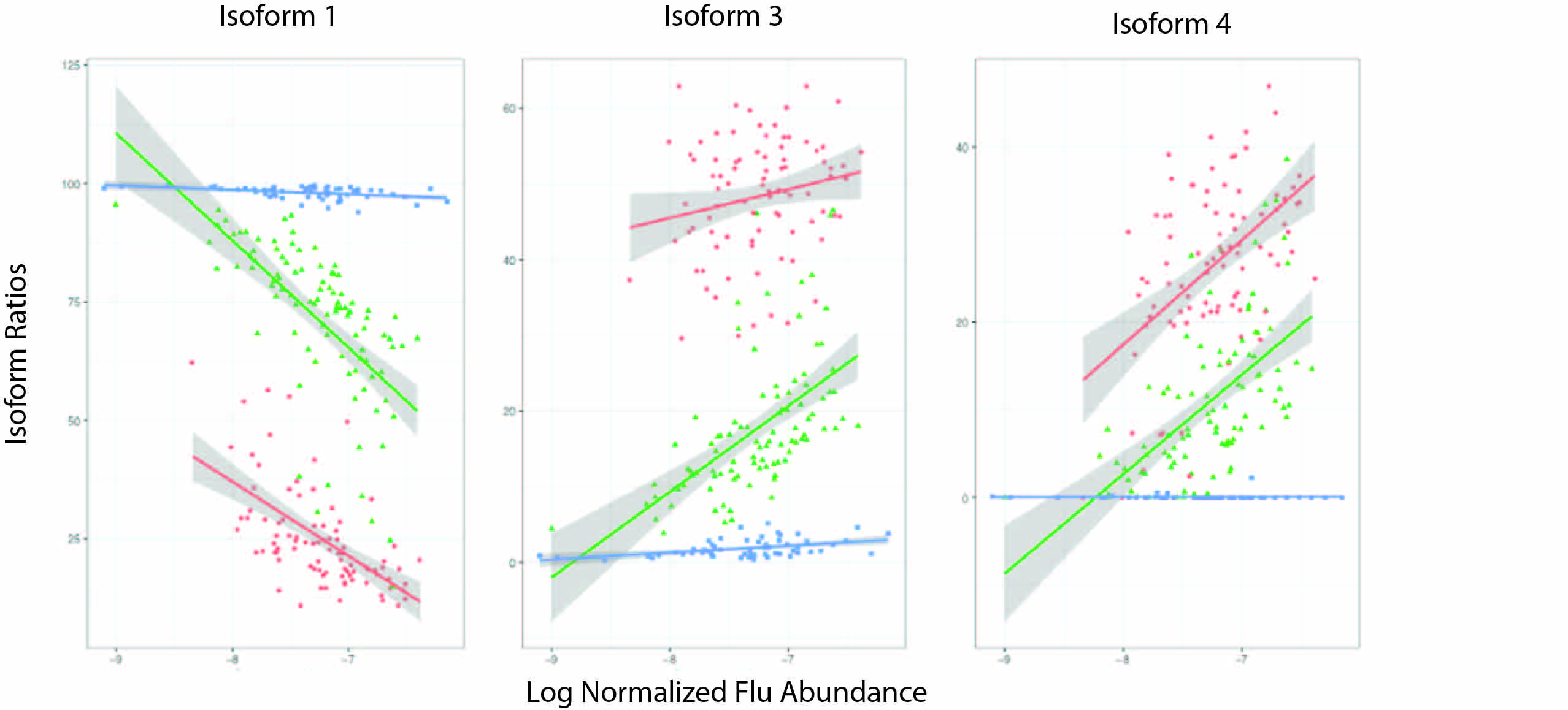
Fig. S17** – Ratio of *ERAP2* isoforms to total *ERAP2* abundance expression (y-axis) vs log normalized flu segment 4 transcript abundance (x-axis). Color and shape indicate rs2248374 genotype (Red: GG, Green: AG, Blue: AA).

**Fig. S18** – RNA-seq reads at *ERAP2* exon10 from H3N2-infected (left) or mock (right) monocyte derived macrophages.

**
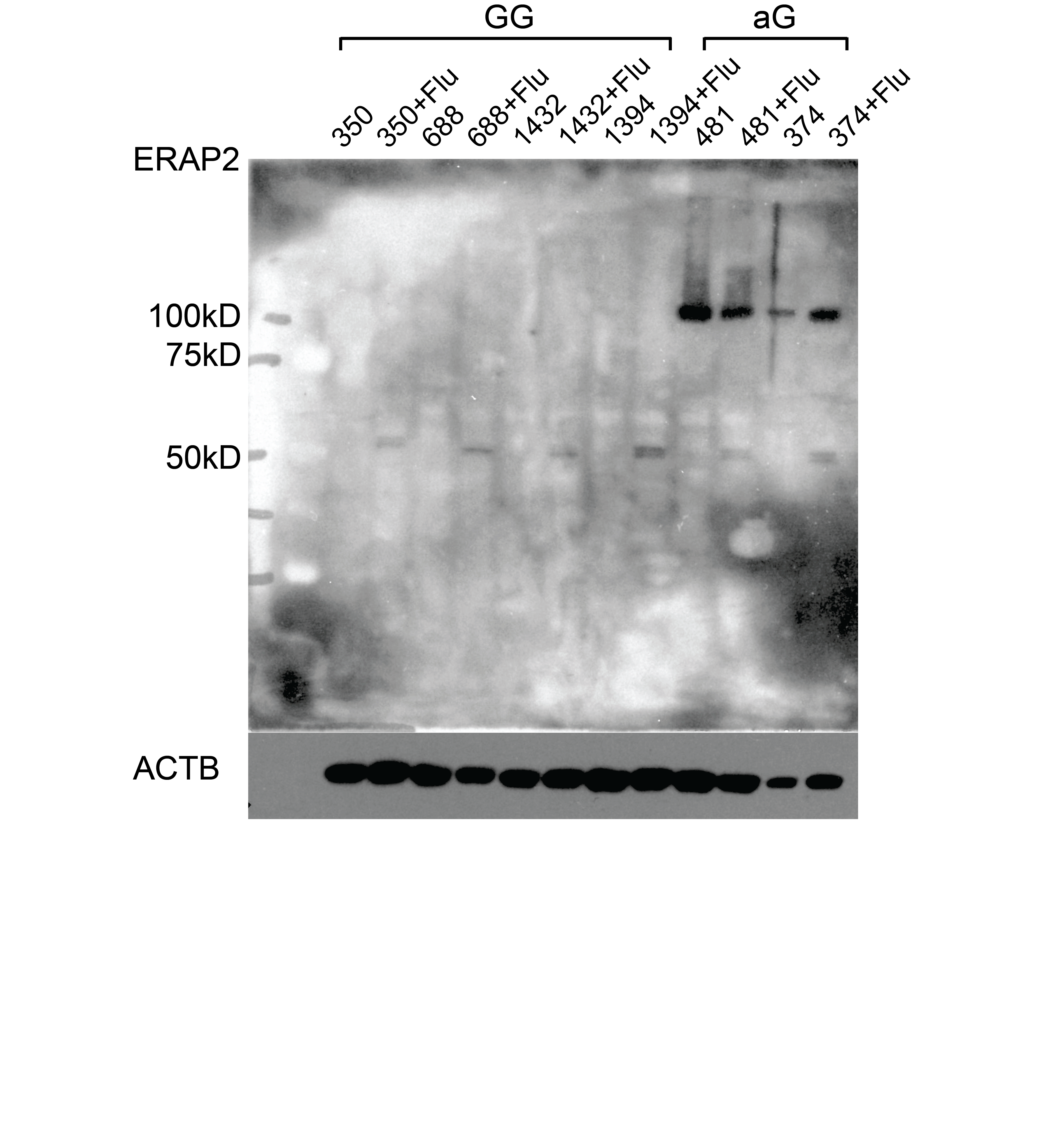
**

**Fig. S19** – Full western blot (see Figure 5D).

**Flu Reference**

>gi|8486138|ref|NC_002023.1| Influenza A virus (A/Puerto Rico/8/34(H1N1)) segment 1, complete sequence

AGCGAAAGCAGGTCAATTATATTCAATATGGAAAGAATAAAAGAACTAAGAAATCTAATGTCGCAGTCTC

GCACCCGCGAGATACTCACAAAAACCACCGTGGACCATATGGCCATAATCAAGAAGTACACATCAGGAAG

ACAGGAGAAGAACCCAGCACTTAGGATGAAATGGATGATGGCAATGAAATATCCAATTACAGCAGACAAG

AGGATAACGGAAATGATTCCTGAGAGAAATGAGCAAGGACAAACTTTATGGAGTAAAATGAATGATGCCG

GATCAGACCGAGTGATGGTATCACCTCTGGCTGTGACATGGTGGAATAGGAATGGACCAATGACAAATAC

AGTTCATTATCCAAAAATCTACAAAACTTATTTTGAAAGAGTCGAAAGGCTAAAGCATGGAACCTTTGGC

CCTGTCCATTTTAGAAACCAAGTCAAAATACGTCGGAGAGTTGACATAAATCCTGGTCATGCAGATCTCA

GTGCCAAGGAGGCACAGGATGTAATCATGGAAGTTGTTTTCCCTAACGAAGTGGGAGCCAGGATACTAAC

ATCGGAATCGCAACTAACGATAACCAAAGAGAAGAAAGAAGAACTCCAGGATTGCAAAATTTCTCCTTTG

ATGGTTGCATACATGTTGGAGAGAGAACTGGTCCGCAAAACGAGATTCCTCCCAGTGGCTGGTGGAACAA

GCAGTGTGTACATTGAAGTGTTGCATTTGACTCAAGGAACATGCTGGGAACAGATGTATACTCCAGGAGG

GGAAGTGAAGAATGATGATGTTGATCAAAGCTTGATTATTGCTGCTAGGAACATAGTGAGAAGAGCTGCA

GTATCAGCAGACCCACTAGCATCTTTATTGGAGATGTGCCACAGCACACAGATTGGTGGAATTAGGATGG

TAGACATCCTTAAGCAGAACCCAACAGAAGAGCAAGCCGTGGGTATATGCAAGGCTGCAATGGGACTGAG

AATTAGCTCATCCTTCAGTTTTGGTGGATTCACATTTAAGAGAACAAGCGGATCATCAGTCAAGAGAGAG

GAAGAGGTGCTTACGGGCAATCTTCAAACATTGAAGATAAGAGTGCATGAGGGATATGAAGAGTTCACAA

TGGTTGGGAGAAGAGCAACAGCCATACTCAGAAAAGCAACCAGGAGATTGATTCAGCTGATAGTGAGTGG

GAGAGACGAACAGTCGATTGCCGAAGCAATAATTGTGGCCATGGTATTTTCACAAGAGGATTGTATGATA

AAAGCAGTTAGAGGTGATCTGAATTTCGTCAATAGGGCGAATCAGCGACTGAATCCTATGCATCAACTTT

TAAGACATTTTCAGAAGGATGCGAAAGTGCTTTTTCAAAATTGGGGAGTTGAACCTATCGACAATGTGAT

GGGAATGATTGGGATATTGCCCGACATGACTCCAAGCATCGAGATGTCAATGAGAGGAGTGAGAATCAGC

AAAATGGGTGTAGATGAGTACTCCAGCACGGAGAGGGTAGTGGTGAGCATTGACCGGTTCTTGAGAGTCC

GGGACCAACGAGGAAATGTACTACTGTCTCCCGAGGAGGTCAGTGAAACACAGGGAACAGAGAAACTGAC

AATAACTTACTCATCGTCAATGATGTGGGAGATTAATGGTCCTGAATCAGTGTTGGTCAATACCTATCAA

TGGATCATCAGAAACTGGGAAACTGTTAAAATTCAGTGGTCCCAGAACCCTACAATGCTATACAATAAAA

TGGAATTTGAACCATTTCAGTCTTTAGTACCTAAGGCCATTAGAGGCCAATACAGTGGGTTTGTGAGAAC

TCTGTTCCAACAAATGAGGGATGTGCTTGGGACATTTGATACCGCACAGATAATAAAACTTCTTCCCTTC

GCAGCCGCTCCACCAAAGCAAAGTAGAATGCAGTTCTCCTCATTTACTGTGAATGTGAGGGGATCAGGAA

TGAGAATACTTGTAAGGGGCAATTCTCCTGTATTCAACTACAACAAGGCCACGAAGAGACTCACAGTTCT

CGGAAAGGATGCTGGCACTTTAACCGAAGACCCAGATGAAGGCACAGCTGGAGTGGAGTCCGCTGTTCTG

AGGGGATTCCTCATTCTGGGCAAAGAAGACAGGAGATATGGGCCAGCATTAAGCATCAATGAACTGAGCA

ACCTTGCGAAAGGAGAGAAGGCTAATGTGCTAATTGGGCAAGGAGACGTGGTGTTGGTAATGAAACGAAA

ACGGGACTCTAGCATACTTACTGACAGCCAGACAGCGACCAAAAGAATTCGGATGGCCATCAATTAGTGT

CGAATAGTTTAAAAACGACCTTGTTTCTACT

>gi|8486136|ref|NC_002022.1| Influenza A virus (A/Puerto Rico/8/34(H1N1)) segment 3, complete sequence

AGCGAAAGCAGGTACTGATCCAAAATGGAAGATTTTGTGCGACAATGCTTCAATCCGATGATTGTCGAGC

TTGCGGAAAAAACAATGAAAGAGTATGGGGAGGACCTGAAAATCGAAACAAACAAATTTGCAGCAATATG

CACTCACTTGGAAGTATGCTTCATGTATTCAGATTTCCACTTCATCAATGAGCAAGGCGAGTCAATAATC

GTAGAACTTGGTGATCCTAATGCACTTTTGAAGCACAGATTTGAAATAATCGAGGGAAGAGATCGCACAA

TGGCCTGGACAGTAGTAAACAGTATTTGCAACACTACAGGGGCTGAGAAACCAAAGTTTCTACCAGATTT

GTATGATTACAAGGAAAATAGATTCATCGAAATTGGAGTAACAAGGAGAGAAGTTCACATATACTATCTG

GAAAAGGCCAATAAAATTAAATCTGAGAAAACACACATCCACATTTTCTCGTTCACTGGGGAAGAAATGG

CCACAAAGGCCGACTACACTCTCGATGAAGAAAGCAGGGCTAGGATCAAAACCAGGCTATTCACCATAAG

ACAAGAAATGGCCAGCAGAGGCCTCTGGGATTCCTTTCGTCAGTCCGAGAGAGGAGAAGAGACAATTGAA

GAAAGGTTTGAAATCACAGGAACAATGCGCAAGCTTGCCGACCAAAGTCTCCCGCCGAACTTCTCCAGCC

TTGAAAATTTTAGAGCCTATGTGGATGGATTCGAACCGAACGGCTACATTGAGGGCAAGCTGTCTCAAAT

GTCCAAAGAAGTAAATGCTAGAATTGAACCTTTTTTGAAAACAACACCACGACCACTTAGACTTCCGAAT

GGGCCTCCCTGTTCTCAGCGGTCCAAATTCCTGCTGATGGATGCCTTAAAATTAAGCATTGAGGACCCAA

GTCATGAAGGAGAGGGAATACCGCTATATGATGCAATCAAATGCATGAGAACATTCTTTGGATGGAAGGA

ACCCAATGTTGTTAAACCACACGAAAAGGGAATAAATCCAAATTATCTTCTGTCATGGAAGCAAGTACTG

GCAGAACTGCAGGACATTGAGAATGAGGAGAAAATTCCAAAGACTAAAAATATGAAAAAAACAAGTCAGC

TAAAGTGGGCACTTGGTGAGAACATGGCACCAGAAAAGGTAGACTTTGACGACTGTAAAGATGTAGGTGA

TTTGAAGCAATATGATAGTGATGAACCAGAATTGAGGTCGCTTGCAAGTTGGATTCAGAATGAGTTCAAC

AAGGCATGCGAACTGACAGATTCAAGCTGGATAGAGCTTGATGAGATTGGAGAAGATGTGGCTCCAATTG

AACACATTGCAAGCATGAGAAGGAATTATTTCACATCAGAGGTGTCTCACTGCAGAGCCACAGAATACAT

AATGAAGGGGGTGTACATCAATACTGCCTTACTTAATGCATCTTGTGCAGCAATGGATGATTTCCAATTA

ATTCCAATGATAAGCAAGTGTAGAACTAAGGAGGGAAGGCGAAAGACCAACTTGTATGGTTTCATCATAA

AAGGAAGATCCCACTTAAGGAATGACACCGACGTGGTAAACTTTGTGAGCATGGAGTTTTCTCTCACTGA

CCCAAGACTTGAACCACACAAATGGGAGAAGTACTGTGTTCTTGAGATAGGAGATATGCTTCTAAGAAGT

GCCATAGGCCAGGTTTCAAGGCCCATGTTCTTGTATGTGAGGACAAATGGAACCTCAAAAATTAAAATGA

AATGGGGAATGGAGATGAGGCGTTGTCTCCTCCAGTCACTTCAACAAATTGAGAGTATGATTGAAGCTGA

GTCCTCTGTCAAAGAGAAAGACATGACCAAAGAGTTCTTTGAGAACAAATCAGAAACATGGCCCATTGGA

GAGTCTCCCAAAGGAGTGGAGGAAAGTTCCATTGGGAAGGTCTGCAGGACTTTATTAGCAAAGTCGGTAT

TTAACAGCTTGTATGCATCTCCACAACTAGAAGGATTTTCAGCTGAATCAAGAAAACTGCTTCTTATCGT

TCAGGCTCTTAGGGACAATCTGGAACCTGGGACCTTTGATCTTGGGGGGCTATATGAAGCAATTGAGGAG

TGCCTAATTAATGATCCCTGGGTTTTGCTTAATGCTTCTTGGTTCAACTCCTTCCTTACACATGCATTGA

GTTAGTTGTGGCAGTGCTACTATTTGCTATCCATACTGTCCAAAAAAGTACCTTGTTTCTACT

>gi|8486134|ref|NC_002021.1| Influenza A virus (A/Puerto Rico/8/34(H1N1)) segment 2, complete sequence

AGCGAAAGCAGGCAAACCATTTGAATGGATGTCAATCCGACCTTACTTTTCTTAAAAGTGCCAGCACAAA

ATGCTATAAGCACAACTTTCCCTTATACCGGAGACCCTCCTTACAGCCATGGGACAGGAACAGGATACAC

CATGGATACTGTCAACAGGACACATCAGTACTCAGAAAAGGCAAGATGGACAACAAACACCGAAACTGGA

GCACCGCAACTCAACCCGATTGATGGGCCACTGCCAGAAGACAATGAACCAAGTGGTTATGCCCAAACAG

ATTGTGTATTGGAAGCAATGGCTTTCCTTGAGGAATCCCATCCTGGTATTTTTGAAAACTCGTGTATTGA

AACGATGGAGGTTGTTCAGCAAACACGAGTAGACAAGCTGACACAAGGCCGACAGACCTATGACTGGACT

TTAAATAGAAACCAGCCTGCTGCAACAGCATTGGCCAACACAATAGAAGTGTTCAGATCAAATGGCCTCA

CGGCCAATGAGTCTGGAAGGCTCATAGACTTCCTTAAGGATGTAATGGAGTCAATGAAAAAAGAAGAAAT

GGGGATCACAACTCATTTTCAGAGAAAGAGACGGGTGAGAGACAATATGACTAAGAAAATGATAACACAG

AGAACAATAGGTAAAAGGAAACAGAGATTGAACAAAAGGAGTTATCTAATTAGAGCATTGACCCTGAACA

CAATGACCAAAGATGCTGAGAGAGGGAAGCTAAAACGGAGAGCAATTGCAACCCCAGGGATGCAAATAAG

GGGGTTTGTATACTTTGTTGAGACACTGGCAAGGAGTATATGTGAGAAACTTGAACAATCAGGGTTGCCA

GTTGGAGGCAATGAGAAGAAAGCAAAGTTGGCAAATGTTGTAAGGAAGATGATGACCAATTCTCAGGACA

CCGAACTTTCTTTGACCATCACTGGAGATAACACCAAATGGAACGAAAATCAGAATCCTCGGATGTTTTT

GGCCATGATCACATATATGACCAGAAATCAGCCCGAATGGTTCAGAAATGTTCTAAGTATTGCTCCAATA

ATGTTCTCAAACAAAATGGCGAGACTGGGAAAAGGGTATATGTTTGAGAGCAAGAGTATGAAACTTAGAA

CTCAAATACCTGCAGAAATGCTAGCAAGCATTGATTTGAAATATTTCAATGATTCAACAAGAAAGAAGAT

TGAAAAAATCCGACCGCTCTTAATAGAGGGGACTGCATCATTGAGCCCTGGAATGATGATGGGCATGTTC

AATATGTTAAGCACTGTATTAGGCGTCTCCATCCTGAATCTTGGACAAAAGAGATACACCAAGACTACTT

ACTGGTGGGATGGTCTTCAATCCTCTGACGATTTTGCTCTGATTGTGAATGCACCCAATCATGAAGGGAT

TCAAGCCGGAGTCGACAGGTTTTATCGAACCTGTAAGCTACATGGAATCAATATGAGCAAGAAAAAGTCT

TACATAAACAGAACAGGTACATTTGAATTCACAAGTTTTTTCTATCGTTATGGGTTTGTTGCCAATTTCA

GCATGGAGCTTCCCAGTTTTGGTGTGTCTGGGAGCAACGAGTCAGCGGACATGAGTATTGGAGTTACTGT

CATCAAAAACAATATGATAAACAATGATCTTGGTCCAGCAACAGCTCAAATGGCCCTTCAGTTGTTCATC

AAAGATTACAGGTACACGTACCGATGCCATAGAGGTGACACACAAATACAAACCCGAAGATCATTTGAAA

TAAAGAAACTGTGGGAGCAAACCCGTTCCAAAGCTGGACTGCTGGTCTCCGACGGAGGCCCAAATTTATA

CAACATTAGAAATCTCCACATTCCTGAAGTCTGCCTAAAATGGGAATTGATGGATGAGGATTACCAGGGG

CGTTTATGCAACCCACTGAACCCATTTGTCAGCCATAAAGAAATTGAATCAATGAACAATGCAGTGATGA

TGCCAGCACATGGTCCAGCCAAAAACATGGAGTATGATGCTGTTGCAACAACACACTCCTGGATCCCCAA

AAGAAATCGATCCATCTTGAATACAAGTCAAAGAGGAGTACTTGAAGATGAACAAATGTACCAAAGGTGC

TGCAATTTATTTGAAAAATTCTTCCCCAGCAGTTCATACAGAAGACCAGTCGGGATATCCAGTATGGTGG

AGGCTATGGTTTCCAGAGCCCGAATTGATGCACGGATTGATTTCGAATCTGGAAGGATAAAGAAAGAAGA

GTTCACTGAGATCATGAAGATCTGTTCCACCATTGAAGAGCTCAGACGGCAAAAATAGTGAATTTAGCTT

GTCCTTCATGAAAAAATGCCTTGTTCCTACT

>gi|8486131|ref|NC_002020.1| Influenza A virus (A/Puerto Rico/8/34(H1N1)) segment 8, complete sequence

AGCAAAAGCAGGGTGACAAAGACATAATGGATCCAAACACTGTGTCAAGCTTTCAGGTAGATTGCTTTCT

TTGGCATGTCCGCAAACGAGTTGCAGACCAAGAACTAGGTGATGCCCCATTCCTTGATCGGCTTCGCCGA

GATCAGAAATCCCTAAGAGGAAGGGGCAGCACTCTTGGTCTGGACATCGAGACAGCCACACGTGCTGGAA

AGCAGATAGTGGAGCGGATTCTGAAAGAAGAATCCGATGAGGCACTTAAAATGACCATGGCCTCTGTACC

TGCGTCGCGTTACCTAACCGACATGACTCTTGAGGAAATGTCAAGGGAATGGTCCATGCTCATACCCAAG

CAGAAAGTGGCAGGCCCTCTTTGTATCAGAATGGACCAGGCGATCATGGATAAAAACATCATACTGAAAG

CGAACTTCAGTGTGATTTTTGACCGGCTGGAGACTCTAATATTGCTAAGGGCTTTCACCGAAGAGGGAGC

AATTGTTGGCGAAATTTCACCATTGCCTTCTCTTCCAGGACATACTGCTGAGGATGTCAAAAATGCAGTT

GGAGTCCTCATCGGAGGACTTGAATGGAATGATAACACAGTTCGAGTCTCTGAAACTCTACAGAGATTCG

CTTGGAGAAGCAGTAATGAGAATGGGAGACCTCCACTCACTCCAAAACAGAAACGAGAAATGGCGGGAAC

AATTAGGTCAGAAGTTTGAAGAAATAAGATGGTTGATTGAAGAAGTGAGACACAAACTGAAGGTAACAGA

GAATAGTTTTGAGCAAATAACATTTATGCAAGCCTTACATCTATTGCTTGAAGTGGAGCAAGAGATAAGA

ACTTTCTCATTTCAGCTTATTTAATAATAAAAAACACCCTTGTTTCTACT

>gi|8486129|ref|NC_002019.1| Influenza A virus (A/Puerto Rico/8/34(H1N1)) segment 5, complete sequence

AGCAAAAGCAGGGTAGATAATCACTCACTGAGTGACATCAAAATCATGGCGTCCCAAGGCACCAAACGGT

CTTACGAACAGATGGAGACTGATGGAGAACGCCAGAATGCCACTGAAATCAGAGCATCCGTCGGAAAAAT

GATTGGTGGAATTGGACGATTCTACATCCAAATGTGCACAGAACTTAAACTCAGTGATTATGAGGGACGG

TTGATCCAAAACAGCTTAACAATAGAGAGAATGGTGCTCTCTGCTTTTGACGAAAGGAGAAATAAATACC

TGGAAGAACATCCCAGTGCGGGGAAGGATCCTAAGAAAACTGGAGGACCTATATACAGAAGAGTAAACGG

AAAGTGGATGAGAGAACTCATCCTTTATGACAAAGAAGAAATAAGGCGAATCTGGCGCCAAGCTAATAAT

GGTGACGATGCAACGGCTGGTCTGACTCACATGATGATCTGGCATTCCAATTTGAATGATGCAACTTATC

AGAGGACAAGGGCTCTTGTTCGCACCGGAATGGATCCCAGGATGTGCTCTCTGATGCAAGGTTCAACTCT

CCCTAGGAGGTCTGGAGCCGCAGGTGCTGCAGTCAAAGGAGTTGGAACAATGGTGATGGAATTGGTCAGG

ATGATCAAACGTGGGATCAATGATCGGAACTTCTGGAGGGGTGAGAATGGACGAAAAACAAGAATTGCTT

ATGAAAGAATGTGCAACATTCTCAAAGGGAAATTTCAAACTGCTGCACAAAAAGCAATGATGGATCAAGT

GAGAGAGAGCCGGGACCCAGGGAATGCTGAGTTCGAAGATCTCACTTTTCTAGCACGGTCTGCACTCATA

TTGAGAGGGTCGGTTGCTCACAAGTCCTGCCTGCCTGCCTGTGTGTATGGACCTGCCGTAGCCAGTGGGT

ACGACTTTGAAAGAGAGGGATACTCTCTAGTCGGAATAGACCCTTTCAGACTGCTTCAAAACAGCCAAGT

GTACAGCCTAATCAGACCAAATGAGAATCCAGCACACAAGAGTCAACTGGTGTGGATGGCATGCCATTCT

GCCGCATTTGAAGATCTAAGAGTATTGAGCTTCATCAAAGGGACGAAGGTGGTCCCAAGAGGGAAGCTTT

CCACTAGAGGAGTTCAAATTGCTTCCAATGAAAATATGGAGACTATGGAATCAAGTACACTTGAACTGAG

AAGCAGGTACTGGGCCATAAGGACCAGAAGTGGAGGAAACACCAATCAACAGAGGGCATCTGCGGGCCAA

ATCAGCATACAACCTACGTTCTCAGTACAGAGAAATCTCCCTTTTGACAGAACAACCGTTATGGCAGCAT

TCACTGGGAATACAGAGGGGAGAACATCTGACATGAGGACCGAAATCATAAGGATGATGGAAAGTGCAAG

ACCAGAAGATGTGTCTTTCCAGGGGCGGGGAGTCTTCGAGCTCTCGGACGAAAAGGCAGCGAGCCCGATC

GTGCCTTCCTTTGACATGAGTAATGAAGGATCTTATTTCTTCGGAGACAATGCAGAGGAGTACGACAATT

AAAGAAAAATACCCTTGTTTCTACT

>gi|8486127|ref|NC_002018.1| Influenza A virus (A/Puerto Rico/8/34(H1N1)) segment 6, complete sequence

AGCGAAAGCAGGGGTTTAAAATGAATCCAAATCAGAAAATAATAACCATTGGATCAATCTGTCTGGTAGT

CGGACTAATTAGCCTAATATTGCAAATAGGGAATATAATCTCAATATGGATTAGCCATTCAATTCAAACT

GGAAGTCAAAACCATACTGGAATATGCAACCAAAACATCATTACCTATAAAAATAGCACCTGGGTAAAGG

ACACAACTTCAGTGATATTAACCGGCAATTCATCTCTTTGTCCCATCCGTGGGTGGGCTATATACAGCAA

AGACAATAGCATAAGAATTGGTTCCAAAGGAGACGTTTTTGTCATAAGAGAGCCCTTTATTTCATGTTCT

CACTTGGAATGCAGGACCTTTTTTCTGACCCAAGGTGCCTTACTGAATGACAGGCATTCAAATGGGACTG

TTAAGGACAGAAGCCCTTATAGGGCCTTAATGAGCTGCCCTGTCGGTGAAGCTCCGTCCCCGTACAATTC

AAGATTTGAATCGGTTGCTTGGTCAGCAAGTGCATGTCATGATGGCATGGGCTGGCTAACAATCGGAATT

TCAGGTCCAGATAATGGAGCAGTGGCTGTATTAAAATACAACGGCATAATAACTGAAACCATAAAAAGTT

GGAGGAAGAAAATATTGAGGACACAAGAGTCTGAATGTGCCTGTGTAAATGGTTCATGTTTTACTATAAT

GACTGATGGCCCGAGTGATGGGCTGGCCTCGTACAAAATTTTCAAGATCGAAAAGGGGAAGGTTACTAAA

TCAATAGAGTTGAATGCACCTAATTCTCACTATGAGGAATGTTCCTGTTACCCTGATACCGGCAAAGTGA

TGTGTGTGTGCAGAGACAATTGGCATGGTTCGAACCGGCCATGGGTGTCTTTCGATCAAAACCTGGATTA

TCAAATAGGATACATCTGCAGTGGGGTTTTCGGTGACAACCCGCGTCCCAAAGATGGAACAGGCAGCTGT

GGTCCAGTGTATGTTGATGGAGCAAACGGAGTAAAGGGATTTTCATATAGGTATGGTAATGGTGTTTGGA

TAGGAAGGACCAAAAGTCACAGTTCCAGACATGGGTTTGAGATGATTTGGGATCCTAATGGATGGACAGA

GACTGATAGTAAGTTCTCTGTGAGGCAAGATGTTGTGGCAATGACTGATTGGTCAGGGTATAGCGGGAGT

TTCGTTCAACATCCTGAGCTAACAGGGCTAGACTGTATAAGGCCGTGCTTCTGGGTTGAATTAATCAGGG

GACGACCTAAAGAAAAAACAATCTGGACTAGTGCGAGCAGCATTTCTTTTTGTGGCGTGAATAGTGATAC

TGTAGATTGGTCTTGGCCAGACGGTGCTGAGTTGCCATTCACCATTGACAAGTAGTCTGTTCAAAAAACT

CCTTGTTTCTACT

>gi|8486125|ref|NC_002017.1| Influenza A virus (A/Puerto Rico/8/34(H1N1)) segment 4, complete sequence

AGCAAAAGCAGGGGAAAATAAAAACAACCAAAATGAAGGCAAACCTACTGGTCCTGTTATGTGCACTTGC

AGCTGCAGATGCAGACACAATATGTATAGGCTACCATGCGAACAATTCAACCGACACTGTTGACACAGTG

CTCGAGAAGAATGTGACAGTGACACACTCTGTTAACCTGCTCGAAGACAGCCACAACGGAAAACTATGTA

GATTAAAAGGAATAGCCCCACTACAATTGGGGAAATGTAACATCGCCGGATGGCTCTTGGGAAACCCAGA

ATGCGACCCACTGCTTCCAGTGAGATCATGGTCCTACATTGTAGAAACACCAAACTCTGAGAATGGAATA

TGTTATCCAGGAGATTTCATCGACTATGAGGAGCTGAGGGAGCAATTGAGCTCAGTGTCATCATTCGAAA

GATTCGAAATATTTCCCAAAGAAAGCTCATGGCCCAACCACAACACAACCAAAGGAGTAACGGCAGCATG

CTCCCATGCGGGGAAAAGCAGTTTTTACAGAAATTTGCTATGGCTGACGGAGAAGGAGGGCTCATACCCA

AAGCTGAAAAATTCTTATGTGAACAAGAAAGGGAAAGAAGTCCTTGTACTGTGGGGTATTCATCACCCGT

CTAACAGTAAGGATCAACAGAATATCTATCAGAATGAAAATGCTTATGTCTCTGTAGTGACTTCAAATTA

TAACAGGAGATTTACCCCGGAAATAGCAGAAAGACCCAAAGTAAGAGATCAAGCTGGGAGGATGAACTAT

TACTGGACCTTGCTAAAACCCGGAGACACAATAATATTTGAGGCAAATGGAAATCTAATAGCACCAAGGT

ATGCTTTCGCACTGAGTAGAGGCTTTGGGTCCGGCATCATCACCTCAAACGCATCAATGCATGAGTGTAA

CACGAAGTGTCAAACACCCCTGGGAGCTATAAACAGCAGTCTCCCTTTCCAGAATATACACCCAGTCACA

ATAGGAGAGTGCCCAAAATACGTCAGGAGTGCCAAATTGAGGATGGTTACAGGACTAAGGAACATTCCGT

CCATTCAATCCAGAGGTCTATTTGGAGCCATTGCCGGTTTTATTGAAGGGGGATGGACTGGAATGATAGA

TGGATGGTACGGTTATCATCATCAGAATGAACAGGGATCAGGCTATGCAGCGGATCAAAAAAGCACACAA

AATGCCATTAACGGGATTACAAACAAGGTGAACTCTGTTATCGAGAAAATGAACATTCAATTCACAGCTG

TGGGTAAAGAATTCAACAAATTAGAAAAAAGGATGGAAAATTTAAATAAAAAAGTTGATGATGGATTTCT

GGACATTTGGACATATAATGCAGAATTGTTAGTTCTACTGGAAAATGAAAGGACTCTGGATTTCCATGAC

TCAAATGTGAAGAATCTGTATGAGAAAGTAAAAAGCCAATTAAAGAATAATGCCAAAGAAATCGGAAATG

GATGTTTTGAGTTCTACCACAAGTGTGACAATGAATGCATGGAAAGTGTAAGAAATGGGACTTATGATTA

TCCCAAATATTCAGAAGAGTCAAAGTTGAACAGGGAAAAGGTAGATGGAGTGAAATTGGAATCAATGGGG

ATCTATCAGATTCTGGCGATCTACTCAACTGTCGCCAGTTCACTGGTGCTTTTGGTCTCCCTGGGGGCAA

TCAGTTTCTGGATGTGTTCTAATGGATCTTTGCAGTGCAGAATATGCATCTGAGATTAGAATTTCAGAAA

TATGAGGAAAAACACCCTTGTTTCTACT

>gi|8486122|ref|NC_002016.1| Influenza A virus (A/Puerto Rico/8/34(H1N1)) segment 7, complete sequence

AGCGAAAGCAGGTAGATATTGAAAGATGAGTCTTCTAACCGAGGTCGAAACGTACGTTCTCTCTATCATC

CCGTCAGGCCCCCTCAAAGCCGAGATCGCACAGAGACTTGAAGATGTCTTTGCAGGGAAGAACACCGATC

TTGAGGTTCTCATGGAATGGCTAAAGACAAGACCAATCCTGTCACCTCTGACTAAGGGGATTTTAGGATT

TGTGTTCACGCTCACCGTGCCCAGTGAGCGAGGACTGCAGCGTAGACGCTTTGTCCAAAATGCCCTTAAT

GGGAACGGGGATCCAAATAACATGGACAAAGCAGTTAAACTGTATAGGAAGCTCAAGAGGGAGATAACAT

TCCATGGGGCCAAAGAAATCTCACTCAGTTATTCTGCTGGTGCACTTGCCAGTTGTATGGGCCTCATATA

CAACAGGATGGGGGCTGTGACCACTGAAGTGGCATTTGGCCTGGTATGTGCAACCTGTGAACAGATTGCT

GACTCCCAGCATCGGTCTCATAGGCAAATGGTGACAACAACCAACCCACTAATCAGACATGAGAACAGAA

TGGTTTTAGCCAGCACTACAGCTAAGGCTATGGAGCAAATGGCTGGATCGAGTGAGCAAGCAGCAGAGGC

CATGGAGGTTGCTAGTCAGGCTAGGCAAATGGTGCAAGCGATGAGAACCATTGGGACTCATCCTAGCTCC

AGTGCTGGTCTGAAAAATGATCTTCTTGAAAATTTGCAGGCCTATCAGAAACGAATGGGGGTGCAGATGC

AACGGTTCAAGTGATCCTCTCGCTATTGCCGCAAATATCATTGGGATCTTGCACTTGATATTGTGGATTC

TTGATCGTCTTTTTTTCAAATGCATTTACCGTCGCTTTAAATACGGACTGAAAGGAGGGCCTTCTACGGA

AGGAGTGCCAAAGTCTATGAGGGAAGAATATCGAAAGGAACAGCAGAGTGCTGTGGATGCTGACGATGGT

CATTTTGTCAGCATAGAGCTGGAGTAAAAAACTACCTTGTTTCTACT
